# Rational design of metal–organic frameworks (MOFs) as hosts for nanoparticles in catalytic applications: concepts, strategies, and emerging trends

**DOI:** 10.1039/d5qi01201e

**Published:** 2025-07-22

**Authors:** Monnaya Chalermnon, Sophie R. Thomas, Jia Min Chin, Michael R. Reithofer

**Affiliations:** a Institute of Inorganic Chemistry, Faculty of Chemistry, University of Vienna Währinger Str. 42 1090 Vienna Austria michael.reithofer@univie.ac.at; b Institute of Functional Materials and Catalysis, Faculty of Chemistry, University of Vienna Währinger Str. 42 1090 Vienna Austria jiamin.chin@univie.ac.at

## Abstract

Metal–organic frameworks (MOFs) are a versatile class of porous coordination materials that have found widespread application in various fields, particularly as heterogeneous catalysts. Due to the modular nature and molecular tunability of the metal node-linker coordination in MOFs, they are considered competent hosts for secondary materials in their extensive pore channels. Modifications of the metal nodes or ligand functionalisation in MOFs can improve the anchoring ability of nanoparticles, effectively enhance the nanoparticles’ stability, and mitigate the inherent nature of nanoparticles to aggregate. In this review, the synthetic strategies (“ship-in bottle”, “bottle-around-ship”, and one-pot) and novel characterisation techniques of nanoparticle-MOF (NP-MOF) composites are discussed in detail. The precise determination of nanoparticle-MOF coordination is crucial to shed light on the structure–activity relationships of the catalytic composites. Recognising the synergistic properties of MOFs and metallic nanoparticles, we also explore recent advancements in NP-MOF composites with a special focus on zirconium-based MOFs for catalytic applications within the last five years. Therefore, we aim to aid the reader in evaluating the up-to-date and state-of-the-art advancements concerning the chemistry of nanoparticles and MOFs as catalysts, acting as a path for future learning and optimisations.

## Abstract 2

Metal–organic frameworks (MOFs) are a versatile class of porous coordination materials that have found widespread application in various fields, particularly as heterogeneous catalysts.

## Graphical abstract

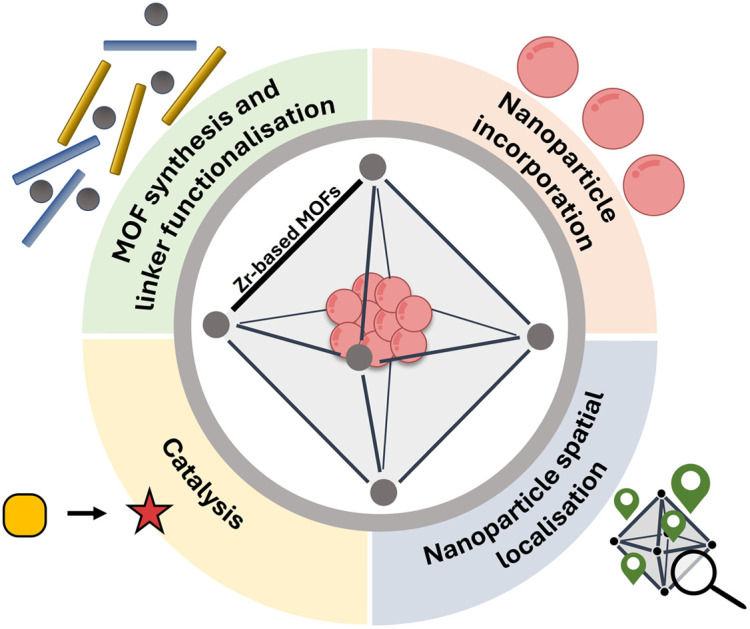

## 1. Introduction

Metal–organic frameworks (MOFs) have received much attention as porous materials due to their versatile properties, including large surface area, well-ordered pores, and the ability to be tailored for specific applications. Since the introduction of the term ‘MOF’ in 1995 by Yaghi *et al*., numerous advancements have been made focusing on modifying the MOF structures by functionalizing the ligands, fine-tuning the metal nodes, or incorporating secondary materials.^1^ The construction of MOFs is relatively straightforward, consisting of organic linkers, known as struts, that connect with metals or metal clusters, known as nodes. Among the different metals used to form the MOF nodes, zirconium (Zr)-based MOFs exhibit exceptional properties. The advantages of Zr-based MOFs include high thermal, mechanical, chemical, and water stability, making them suitable for a wide range of applications, including catalysis, gas/contaminants adsorption, and separation. Several Zr-based MOFs have been reported in the literature, with the most commonly studied being UiO-66,^2–5^ UiO-67,^6^ MOF-808,^7^ PCN-222,^8,9^ and NU-1000.^10–17^

Metallic nanoparticles (MNPs) are known for their catalytic activities for various chemical reactions, primarily due to their electronic properties and high surface area. However, a significant limiting factor affecting their performance is their tendency for aggregation. The integration of nanoparticles with MOFs presents a promising solution, giving rise to a new type of heterogeneous catalyst that alleviates aggregation and potentially enhances catalytic activity. Given the extensive catalytic applications of MOFs, we noticed a lack of comprehensive reviews focusing on recent advancements in Zr-based MOFs in combination with MNPs. Therefore, in this review, we start by describing various known synthesis methods that allow for the inclusion of MNPs with MOFs and the influence of MOF functional groups on nanoparticle formation. Since determining the spatial localisation of the nanoparticles in the composite is important to understand structure–property relationships, we also delve into different localisation techniques, as well as the effect of nanoparticle localisation on their catalytic activities. We then focus on recent applications of nanoparticle-incorporated MOFs, with a special focus on Zr-based MOFs as catalysts for CO_2_ conversion and organic reactions.

## 2. Synthesis methods of NP-MOFs

**Fig. 1:**
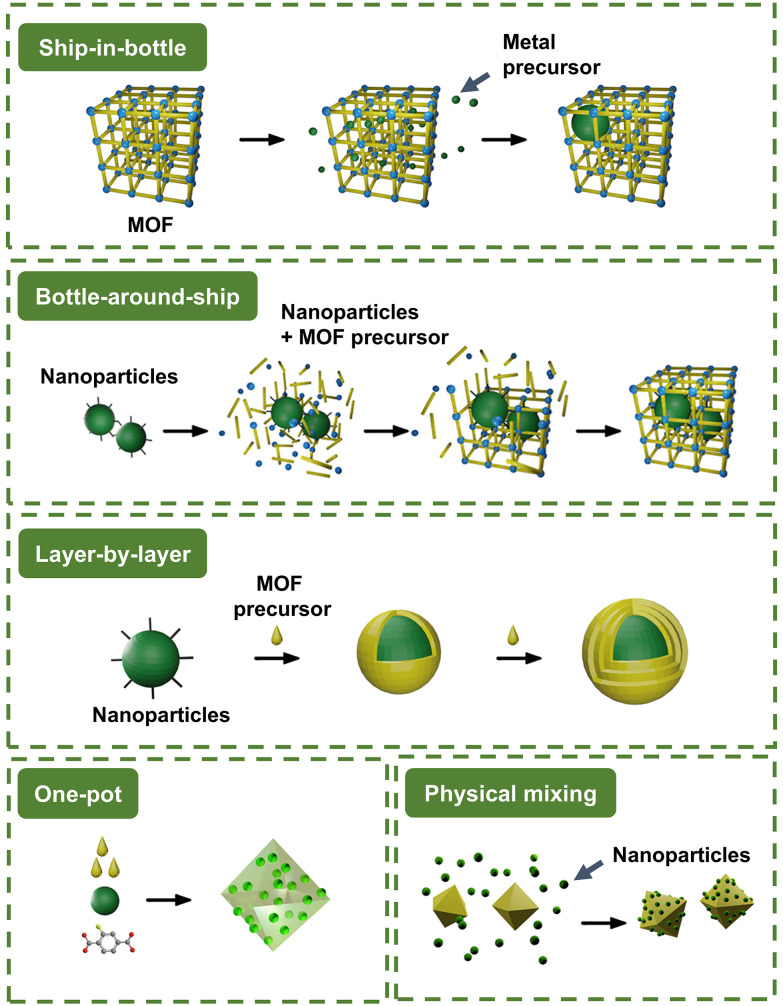
Schematic diagram of the different synthetic methods to form a nanoparticle-MOF composite: “ship-in-bottle” strategy, “bottle-around-ship” strategy, “layer-by-layer” strategy, “one-pot” strategy, and physical mixing strategy. Adapted from ref. 19, Copyright 2022, with permission from Elsevier.

When MOFs are used as hosts for metal nanoparticles, the nanoparticles can either be decorated on the surface (“NP/MOF”) or embedded within the MOF structure (“NP@MOF”). The latter is generally preferred as nanoparticles on the surface may block MOF pores, thus reducing their catalytic performance.^18^ The diversity in the available methodologies for embedding nanoparticles within MOFs offers unique advantages and limitations based on the desired nanoparticle size, distribution, and catalytic performance. The synthesis of NP-MOF composites branches into five categories, as shown in Fig. 1.^19^ Below, the updated synthesis routes for NP-MOF composites *via* “ship-in-bottle”, “bottle-around-ship”, and one-pot synthesis are discussed, detailing the different means to coordinate and localise nanoparticles in the desired area of the Zr-MOFs. We also included the crucial techniques that have been implemented with other metal-based MOFs to expand the interests of the readers.

### 2.1. The “ship-in-bottle” approach

For the “ship-in-bottle” approach, MOFs serve as a template for nanoparticle growth. Typically, this method begins with the addition of metal precursor or synthesised nanoparticles into the MOFs *via* impregnation, deposition, microwave heating, or physical mixing. Then, the metal precursors undergo a reduction process, typically using chemical reducing agents such as sodium borohydride (NaBH_4_) and borane *tert*-butylamine (^*t*^Bu–NH_2_BH_3_). Another reduction method employs a reducing H_2_ atmosphere, where H_2_ can form hydrogen radicals that serve as the reducing agent.^20^ The process is often conducted in the solvent phase, but gas-phase reactions are also possible, albeit used less frequently. A challenge with this approach is controlling the spatial distribution of nanoparticles within the MOF since multiple steps are required. Several examples of monometallic or bimetallic nanoparticle loaded Zr-MOFs have been prepared using this method, for example, Ag/Pd@UiO-66–NH_2_,^21^ UiO-66@Pd,^22^ AgPd@UiO-66–NH_2_, and AgPd@UiO-66–NO_2_.^23^

Additionally, Rosado *et al*. synthesised NU-1000–NH_2_/3-mercaptopropionic acid (PrSH)–copper nanoparticles (CuNPs) using this method.^24^ In detail, the NU-1000–NH_2_ was post-synthetically modified with PrSH followed by impregnation with copper formate hydrate, which binds to the thiol group, before the thermal reduction of Cu at 160 °C under diluted H_2_ in Ar atmosphere at 150 mL min^−1^. The successful formation of CuNPs was confirmed by X-ray photoelectron spectroscopy (XPS) and transmission electron microscopy (TEM) analysis, where binding energy peaks characteristic of Cu^1+^/Cu^0^ were exhibited, and the CuNPs observed were homogeneously distributed throughout the MOF structure.

**Fig. 2:**
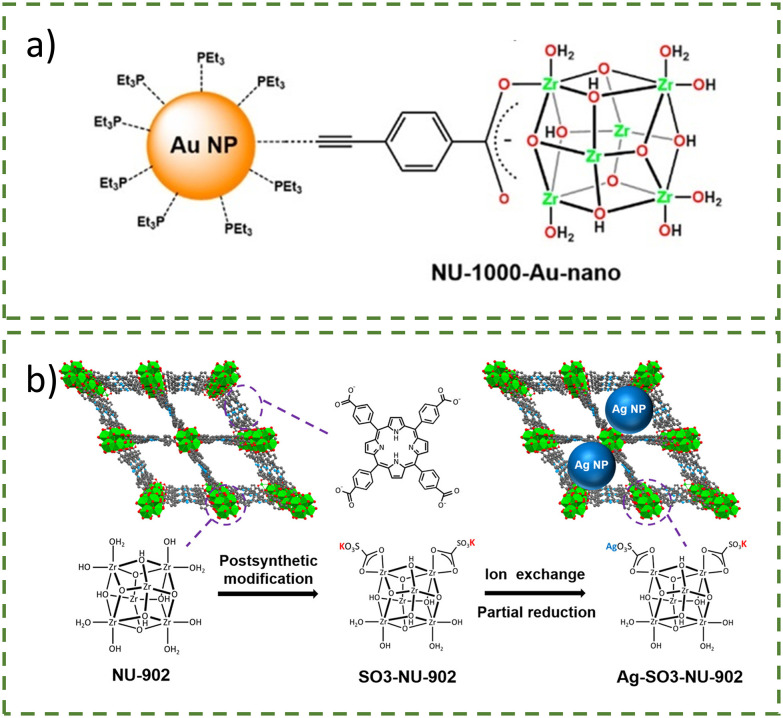
(a) Structure of NU-1000–Au-nano (NU-1000 = [Zr_6_(μ_3_-O)_4_(μ_3_-OH)_4_(OH)_4_(H_2_O)_4_(TBAPy)_2_]_∞_) where the AuNP is bound to the MOF *via* the acetylene group of the carboxy-phenylacetylene linkers. Reprinted with permission from ref. 12. Copyright 2025 American Chemical Society. (b) Schematic representation of the synthesis route of Ag–SO_3_–NU-902. Reprinted (adapted) with permission from ref. 25. Copyright 2020 American Chemical Society.

Similarly, a three-step post-synthetic modification (PSM) was used to create NU-1000–Au-nano, a catalyst for the hydrogenation of 4-nitrophenol (Fig. 2a).^12^ The non-structural carboxy-phenylacetylene (PA) was successfully functionalised onto NU-1000 by coordination of 2.6 units of carboxy-phenylacetylene per Zr cluster *via* the carboxy group (Fig. 2). The acetylene functionality then served as an anchor for the monometallic Au(i)PEt_3_^+^ cation (PEt_3_ = triethylphosphine), a gold precursor, due to the carbophilicity of gold, with 1.4 ± 0.2 Au atoms/Zr_6_ node. Without this acetylene modification, gold installation was minimal at only ∼0.04 Au atoms/Zr_6_ node. Adding a reducing agent (NaBH_4_) to form gold nanoparticles (AuNPs) could be observed visually due to a distinct colour change from yellow to dark brown, indicating AuNP formation. The size of the AuNPs was determined to be 1.5 nm by pair distribution function (PDF) analysis of total X-ray scattering and directed energy deposition (DED), allowing the AuNPs to fit into the triangular pore of NU-1000. This successful encapsulation of AuNPs inside NU-1000 was possible due to the substantial London dispersion force between the linker and the node. Additionally, the affinity of PEt_3_ for AuNPs prevented nanoparticle growth beyond the MOF channel by limiting the migration of gold towards the MOF surface.

Similar to the previous example, Wang *et al*. reported the preparation of silver nanoparticles (AgNPs) confined in SO_3_–NU-902 as electrocatalysts for the oxidation of nitrite (Fig. 2b).^25^ PSM of NU-902 containing a redox-active porphyrinic linker was conducted *via* solvent-assisted ligand incorporation (SALI, see section 3 for more details) with a 4-sulfobenzoic acid potassium salt to yield SO_3_–NU-902. Through an ion exchange reaction with AgBF_4_ in the dark, the reduction of the Ag(i) ions into Ag(0) and immobilisation of Ag(i) inside the MOF pores occurred, yielding Ag–SO_3_–NU-902. XPS confirmed the presence of two oxidation states of silver (Ag(i) and Ag(0)), and TEM showed AgNPs of around 3.4 nm. It has been reported before that it is possible to produce metallic nanoparticles without a reducing agent, and the authors here attributed this phenomenon to the presence of aqua or hydroxo ligands coordinated on the hexa-zirconium nodes of NU-902.

The double solvent method (DSM) exploits solvents of differing polarities to localise metal precursors inside MOF pores. Sun *et al*. combined DSM and photoreduction to encapsulate palladium nanoclusters (PdNCs) (smaller than 1.2 nm) into UiO-66–NH_2_, yielding Pd@NH_2_–UiO-66(Zr) as the catalyst for visible-light-promoted Suzuki coupling reaction.^26^ The localisation of a Pd precursor was achieved by mixing an excess amount of non-polar *n*-hexane with an aqueous solution of Pd(NO_3_)_2_·2H_2_O. This solvent ratio ensured the hydrophilic solution was driven into the MOF pores *via* capillary forces. TEM images confirmed the confinement of Pd nanoclusters, and inductively coupled plasma optical emission spectroscopy (ICP-OES) was used to quantitatively analyse the 0.67 wt% Pd.

Other examples using DSM to encapsulate PdNPs include Pd@UiO-66 for toluene oxidation,^27^ Pd@NH_2_–UiO-66(Zr) as a catalyst for the one-pot synthesis of secondary amines,^28^ Pd@UiO-66–NH_2_@ZnIn_2_S_4_ with a flower-like structure acting as a photocatalyst for hydrogen production,^29^ Pd_*x*_Au_1−*x*_/UiO-66-D for dehydrogenation of formic acid,^30^ and Pd@PCN-222 for hydrogen peroxide (H_2_O_2_) detection.^31^

**Fig. 3:**
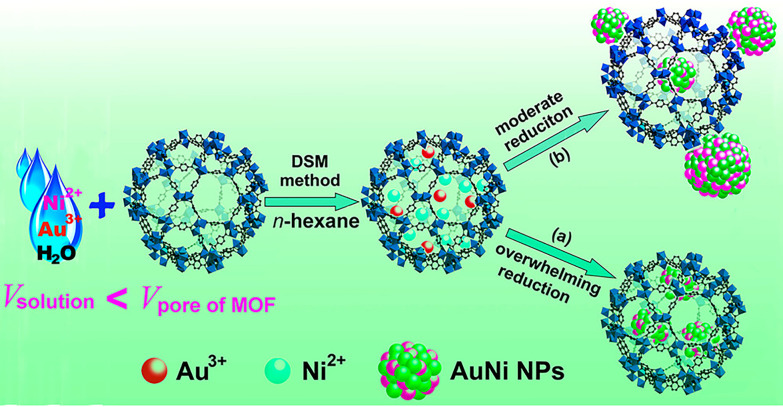
Schematic diagram of the double solvent method (DSM) for the immobilisation of AuNi nanoparticles on a Cr–MIL-101 matrix. (a) Overwhelming reduction is achieved using an excess of reducing agent compared to the MOF pore volume to achieve full reduction and small nanoparticles. (b) Moderate reduction is the result of a lower reductant concentration, resulting in agglomeration and larger nanoparticles due to the ability of the nanoparticles to dissolve and leach to the surface. Reprinted with permission from ref. 32. Copyright 2013 American Chemical Society.

To refine DSM, the liquid-phase concentration-controlled reduction (CCR) strategy allows for controlled synthesis and localisation of nanoparticles as reported by Zhu *et al*.^32^ Electron tomographic reconstruction showed that uniformly distributed and monodispersed Au nickel (Ni) NPs were encapsulated into Cr–MIL-101 using DSM, followed by a so-called ‘overwhelming reduction’ approach (Fig. 3a). In detail, MIL-101 was suspended in *n*-hexane while the aqueous metal precursor was added dropwise to the solution. The *n*-hexane-to-water ratio was kept in a high excess of 100 : 1 to ensure the small amount of aqueous metal precursor solution had a higher tendency to go into the hydrophilic pore. The subsequent overwhelming reduction was accomplished by using 0.6 M NaBH_4_, the concentration of reductant in excess of the MOF pore volume required to completely reduce the loaded metal precursors to ultrafine nanoparticles. When the reductant concentration was lower than the threshold (moderate reduction, Fig. 3b), the metal precursor could redissolve and leach towards the external surface, causing agglomeration and formation of larger nanoparticles.^32^

Chen *et al*. compared the synthesis of Ni@MOF-545 composite using either the impregnation (IM) or double solvent (DS) methods coupled with metal reduction *via* reductive H_2_ atmosphere.^33^ Two types of MOF-545 were used: pristine MOF-545 with free porphyrin linkers and MOF-545 with Cu-metalated porphyrin (‘MOF-545(Cu)’). After impregnating MOF-545 and MOF-545(Cu) with nickel(ii) nitrate dihydrate, the NiNPs were successfully synthesised with both composites containing 37.6 and 35.9 wt% of Ni, respectively, according to ICP-atomic emission spectroscopy (AES) analysis. Interestingly, the reduced sample containing NiNPs showed higher catalytic activity for CH_4_ production compared to the non-reduced sample, despite the nanoparticle formation conditions. This resulted in the amorphisation of the MOF framework, as determined by powder X-ray diffraction (PXRD). Moreover, the presence of Cu did not exhibit additional effects on the catalytic CO_2_ methanation reaction. As an alternative to IM, the DS method was implemented to improve Ni loading. Unfortunately, despite the advantageous capillary force of the DS method, only 23 wt% Ni loading was achieved, and the crystallinity and porosity of the Ni@MOF composite were compromised. Nevertheless, the lower Ni loading of the DS method resulted in a four-fold enhancement of the catalytic CO_2_ methanation compared to the IM-obtained Ni@MOF-545. This improvement was attributed to the smaller (5–6 nm) and more highly dispersed NiNPs on the surface and within the bulk solid of the DS-obtained Ni@MOF-545, as observed by TEM. Additionally, the DS method minimised the Ni deposition on the outer surface, unlike the IM method, which yielded larger NiNPs (30–40 nm) exclusively on the external surfaces of the MOF.^33^

**Fig. 4:**
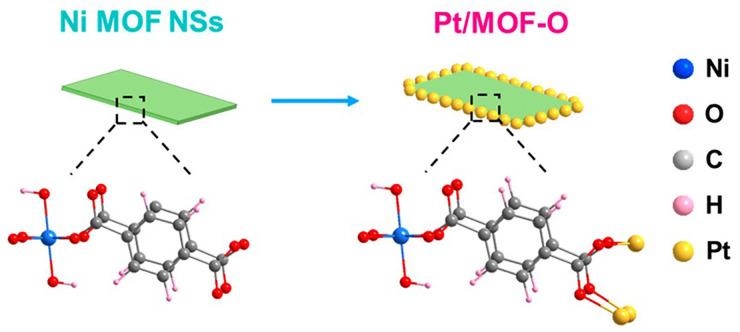
Schematic representation of site-specific growth of PtNPs on the edge of two-dimensional (2D) heterojunction Ni-MOFs using ethylene glycol (EG) or diethyl glycol (DEG) as reducing agent. Reprinted with permission from ref. 34. Copyright 2021 American Chemical Society.

On top of the above-mentioned metalation methods, site-specific nanoparticle growth is also possible by controlling the reduction kinetics of metals with solvents. Wang *et al*. demonstrated site-specific growth of PtNPs on a two-dimensional (2D) heterojunction Ni-MOF (Fig. 4).^34^ When the Ni-MOF was mixed with metal precursors (Pt, Pd, Ag, and Au) in the presence of glycol (a weaker reducing agent) or alcohol (a stronger reducing agent), Pt/MOF-O and Pt/MOF-C were obtained, respectively. When ethylene glycol (EG) or diethyl glycol (DEG) was used as the reducing agent, 1.5 nm PtNPs were localised on the edges of Ni-MOF (Pt/MOF-O) through the coordination to MOF *via p*-phthalic acid, as shown by TEM analysis. In contrast, when stronger reducing agents (methanol or ethanol) were used, larger PtNPs (35 nm) grew randomly on the Ni MOF surface (Pt/MOF-C). Unsurprisingly, the location and size of the PtNPs influenced the catalytic ability of the NP-MOF composite, as evidenced by the hydrogen evolution reaction (HER), with Pt/MOF-O showing superior activity due to the interaction between the PtNPs and MOF through the electron transfer between Pt–O.^34^ On a similar note, Liu *et al*. prepared UiO-67 nanosheets with AuNPs formed *via in situ* reduction of Au(iii) into Au(0) using NaBH_4_. This material was used as a catalyst for the reduction of 4-nitrophenol to 4-aminophenol. Due to the nanosheet structure, the diffusion of the reactant was increased when compared to AuNPs decorated UiO-67 octahedral microcrystals.^35^

**Fig. 5:**
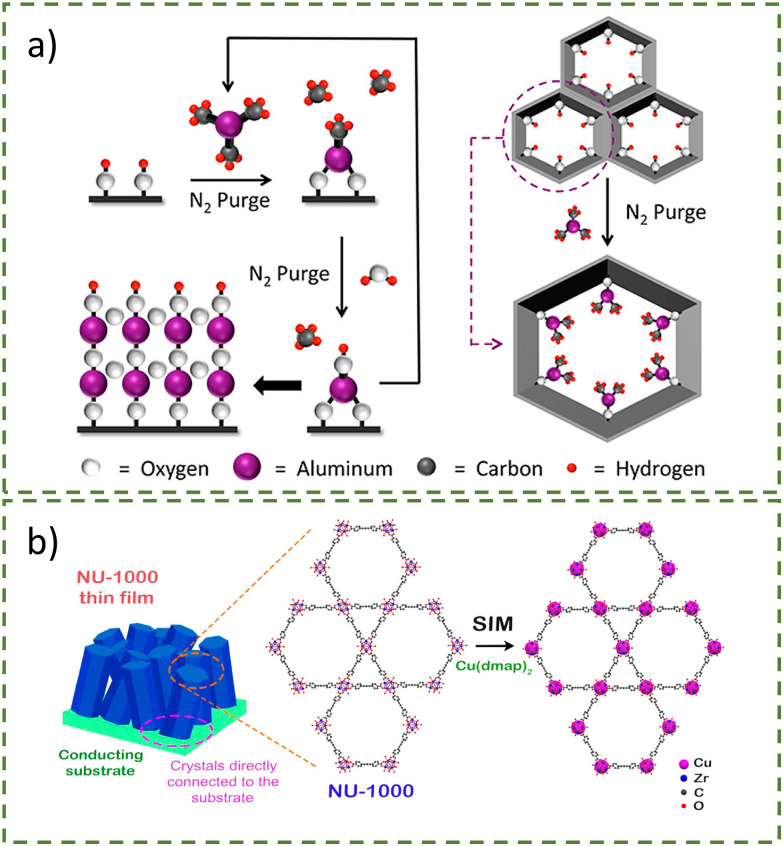
(a) Schematic representation of the vapour-phase atomic layer deposition (ALD) in MOFs (AIM). Reprinted with permission from ref. 37. Copyright 2013 American Chemical Society. (b) Schematic representation of the solvothermal deposition in MOFs (SIM). Reprinted with permission from ref. 38. Copyright 2017 American Chemical Society.

Whilst the metalation process is typically solvent-based, excess solvent molecules can occupy the pores of the MOF or bind to the free metal cluster, potentially hindering the metal deposition process or even the catalytically active sites.^36^ Platero-Prats *et al.* post-synthetically modified NU-1000 *via* vapour-phase atomic layer deposition (ALD) in MOF (AIM). Briefly, the MOF is placed in a chamber at 110 °C to remove physiosorbed water molecules, then the sample is exposed to pulses of the metal precursors.^11^ NU-1000 was chosen for this study due to its stability at high temperatures, mesoporous channels, and spatially oriented hydroxy groups capable of metal coordination. Vapour-phase ALD of NU-1000 with trimethylaluminium (AlMe_3_) or diethylzinc (ZnEt_2_) occurred in an ALD reactor at 120 or 140 °C, respectively, yielding Al-AIM (eight Al atoms per Zr_6_ node) and Zn-AIM (three Zn atoms per Zr_6_ node) (Fig. 5a).^37^ Despite the rapid reaction time and the nature of ALD being a self-limiting reaction that only deposits on surface active areas, the metal loading was lower than that of the solvent-based method, solvothermal deposition in MOF (SIM) (Fig. 5b). Redfern *et al*. improved on this by synthesising Cu@NU-1000 *via* SIM using bis(dimethylamino-2-propoxy)copper(ii) (Cu(dmap)_2_) as the copper precursor.^10,11,38^ They achieved higher copper loading at almost six Cu per Zr_6_ node as compared to four Cu for Cu-AIM, leading to a more effective catalyst for the semihydrogenation of acetylene.

Beyond the common chemical reduction processes, photochemical reduction offers an alternative method for nanoparticle formation. The use of a Xenon lamp is becoming more popular for the reduction of nanoparticles, for example, in 2025, Chen *et al*. developed a AuCu–NU-1000 photocatalyst for CO_2_ reduction into ethane.^39^ The authors proposed a photo-deposition method by first impregnating NU-1000 with AuCl_3_ and CuCl_2_ solutions as gold and copper precursors, respectively, followed by reduction with a 300 W Xenon lamp for one hour under stirring. The difference in reduction potential of gold and copper resulted in a 10-fold increase in gold deposition compared to copper. High-angle annular dark-field scanning electron microscopy (HAADF-STEM) and energy-dispersive X-ray spectroscopy (EDS) mapping of the nanoparticles distributed along NU-1000 showed Cu isolated sites and Cu atomically doped gold supported on NU-1000. Using this method, multiple active sites for photocatalysis have been established. XPS analysis confirmed a Au(0) oxidation state, while copper existed as Cu(0/I) mixed oxidation states. Moreover, Ag/MOF reported by Che *et al*. also used blue LED irradiation (*λ* = 450 nm, 32 W) to obtain AgNPs from impregnated AgNO_3_ in Zr-MOF nanocrystals under an inert atmosphere.^40^ It was found that increasing the duration of irradiation resulted in larger AgNP size (3.1 to 8.6 nm), with higher loading (0.74 wt% (Ag/MOF-5) to 1.96 wt% (Ag/MOF-30)) as determined by TEM and ICP-AES. Ag/MOF-10 synthesised with 10 minutes of irradiation, was the most active catalyst for aerobic cross-dehydrogenative coupling.

### 2.2. The “bottle-around-ship” approach

For the “bottle-around-ship” approach, MOF assembly occurs around pre-synthesised and ligand-stabilised nanoparticles. This allows for a more controlled spatial localisation of nanoparticles; nonetheless, the pre-synthesised nanoparticles may require surfactant/capping agents, which could exceed the size of MOF pores. As the capping/stabilising ligands can detach and either reattach to the nanoparticles or disperse in the system, the choice of capping ligand has a crucial influence on the overall stability and catalytic efficiency of both nanoparticles and MOFs.^12,41^

**Fig. 6:**
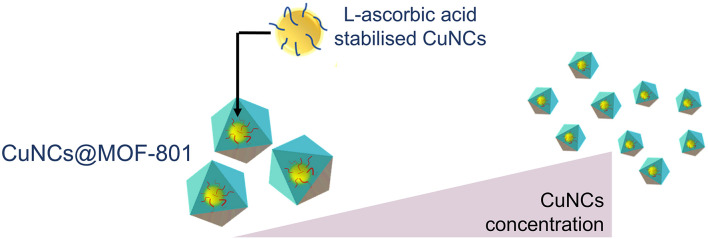
The dependence of particle size of CuNCs@MOF-801, synthesised *via* concentration-dependent seed-mediated synthesis. Adapted with permission from ref. 42. Copyright 2022 American Chemical Society.

Dai *et al*. introduced a novel method to encapsulate ultrasmall CuNCs into MOF-801 or UiO-66–NH_2_.^42^ In this method, CuNCs were pre-synthesised using l-ascorbic acid as the reducing and stabilising agent due to the high affinity of its hydroxy groups to coordinate with Cu. This was followed by the formation of the MOF around the CuNCs (Fig. 6). The authors emphasised the importance of using a weakly acidic synthesis condition due to the susceptibility of CuNCs towards dissolution; therefore, pre-synthesised Zr_6_ oxo clusters were used instead of the more acidic zirconium salt typically used in the conventional solvo/hydrothermal reactions. As a result of the reaction conditions, CuNCs@MOFs with a core–shell arrangement were obtained due to the larger pre-synthesised 1.6 nm CuNCs compared to the MOF pores, which acted as the seeds for seed-mediated MOF growth. The CuNCs exhibited a downsizing effect on the resulting CuNCs@MOFs, where higher CuNC loading led to smaller composite particles, as observed by scanning electron microscopy (SEM) and TEM. ICP-mass spectrometry (MS) analysis showed Cu loadings of 2.8% and 2.5% for CuNCs@MOF-801 and CuNCs@UiO-66–NH_2_, respectively, corresponding to the value obtained from the EDS calculation of the Cu : Zr atomic ratio. Overall, this method allowed for facile gram-scale synthesis of MOF composite, potentially improving the catalytic activity of the composite by preventing partial copper deposition on the outer surface of the MOF.^42^

**Fig. 7:**
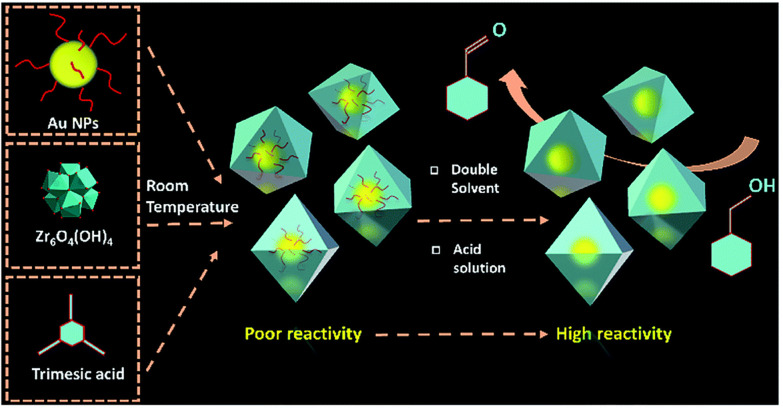
Schematic diagram for the assembly of AuNPs@MOF-808 composites using PVP stabilised AuNPs, Zr_6_O_4_(OH)_4_ and trimesic acid by DSM at room temperature. The PVP removal from the AuNPs by acid treatment resulted in enhanced catalytic activity for the reduction of benzyl alcohol to benzaldehyde. Reprinted with permission from ref. 43. Copyright 2022 Royal Society of Chemistry.

In the same year, the authors further investigated the “bottle-around-ship” method for AuNPs@MOF-808 core–shell composites with an emphasis on improving the photocatalytic activity by removing the capping agent (Fig. 7).^43^ The removal of polyvinylpyrrolidone (PVP) was conducted after AuNPs@MOF-808 synthesis *via* DSM, by treating the sample with 2 M HCl in an acetone/water solvent system. After PVP removal, the catalytic activity was enhanced, achieving 92% conversion of benzyl alcohol to the corresponding aldehyde. On the other hand, the sample before PVP removal exhibited no reactivity.^43^

**Fig. 8:**
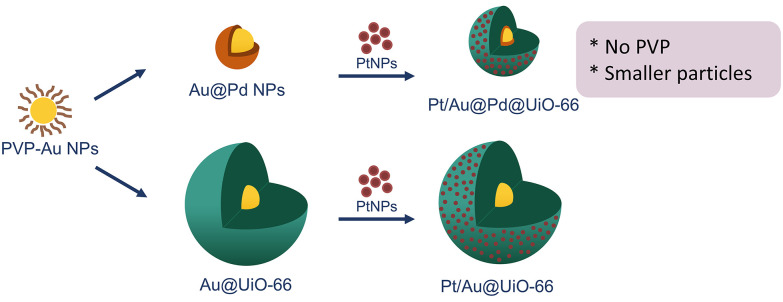
Schematic representation of the two synthetic routes taken to centrally encapsulate AuNPs or Au@PdNPs with uniform dispersion of PtNPs, resulting in Pt/Au@Pd@UiO-66 and Pt/Au@UiO-66 for gas-phase CO_2_ hydrogenation through RWGS. Adapted with permission from ref. 44. Copyright 2018 John Wiley and Sons.

The synthesis of core–shell UiO-66 nanocomposites containing Pt, Pd, and Au as the catalyst for CO_2_ hydrogenation was reported by Zheng *et al*. (Fig. 8).^44^ In this study, a variety of UiO-66 nanocomposites were obtained by mixing pre-synthesised AuNPs (16 nm) or core–shell Au@PdNPs (17 nm) with reactants for UiO-66. When AuNPs were used as the core of the nanocomposite, PVP was required to prevent aggregation and oxidative etching of the AuNPs since the PVP facilitates NP adsorption onto the MOF by the coordination interaction with the carboxy group of the BDC (BDC = 1,4-benzenedicarboxylate) and the Zr^2+^ ion, as well as the hydrophobic interaction between the PVP and BDC.^45^ On the other hand, the presence of Pd eliminated the need for PVP to stabilise the Au@PdNPs and resulted in smaller Au@Pd@UiO-66 (114 ± 11 nm) compared to the parent UiO-66 (409 ± 18 nm) and Au@UiO-66 (405 ± 23 nm).^44^ The final step was the surface deposition of PtNPs, which was achieved by adding an ethanolic solution of pre-synthesised PtNPs with the initial nanocomposites. According to the TEM analysis, PtNPs were homogenously dispersed on the UiO-66 with no aggregation. Overall, the reported stepwise synthesis was capable of controlling the spatial distribution of nanoparticles in MOFs. The distribution of the bimetallic core and the surface PtNPs were beneficial for the CO_2_ conversion reaction, exhibiting the best activity when Pt, Au, and Pd metals were all present.^44^

In a subsequent study, the authors showed that it was feasible to add an additional layer, making a ‘sandwich-like’ structure. With Au@PdNPs as the core, followed by a layer of UiO-67/Pt and enclosing the whole particles with a final UiO-*n* (*n* = 66, 67, 69) layer.^46^ This sandwich structure allowed for the regulation of the reverse water–gas shift (RWGS) reactions.

**Fig. 9:**
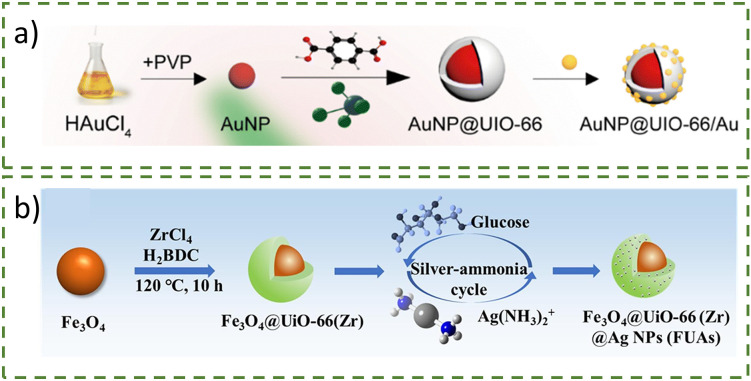
Schematic diagram of the fabrication of (a) AuNP@UiO-66/Au nanoparticles^49^ and (b) Fe_3_O_4_@UiO-66(Zr)@AgNPs.^50^ Reprinted with permission from ref. 49. Copyright 2023 Royal Society of Chemistry. Reprinted with permission from ref. 50. Copyright 2023, with permission from Elsevier.

In addition, core–shell structures have also been shown for nanoparticle-templated MOF growth, which could be further decorated again with more nanoparticles. Due to the plasmonic properties of gold and silver nanoparticles, it is possible to use them to enhance the plasmonic response for surface-enhanced Raman spectroscopy (SERS) analysis to detect traces or degradations of organic compounds.^47,48^ Wu *et al*. reported core–shell AuNP@UiO-66/Au nanoparticles merging the localised surface plasmon resonance (LSPR) feature with catalytic activities into a single platform (Fig. 9a).^49^ The UiO-66 shell was formed onto 50 nm PVP-stabilised gold nanoparticles by reacting the nanoparticle suspension with MOF precursors. The additional 4 nm gold nanoparticles were synthesised by reacting AuNP@UiO-66 with H[AuCl_4_] and reduced with NaBH_4_, yielding AuNP@UiO-66/AuNPs. The UiO-66 shell and small AuNPs acted as the ‘hot spots’ for SERS enhancements. Similarly, the core is not restricted to coinage metal nanoparticles, as shown by Lv *et al*. using around 340 nm Fe_3_O_4_ with a UiO-66 shell (thickness of 16 nm) (Fig. 9b).^50^ The uniformly distributed AgNPs were synthesised through the silver ammonia cycling reaction to obtain the decorated 30 nm AgNPs.

**Fig. 10:**
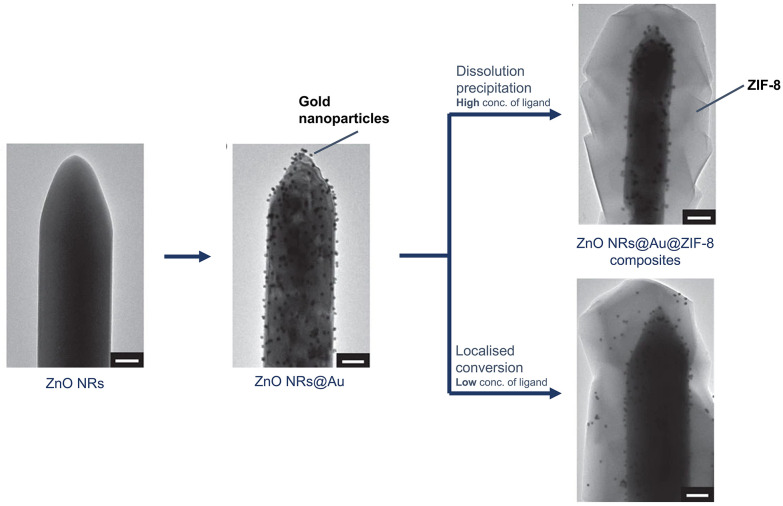
Schematic representation for the spatial regulation using the sacrificial-template synthetic method for the surface modification. TEM images of ZnONRs, ZnONRs@AuNPs, and ZnONRs@AuNPs@ZIF-8 composites with AuNPs at different locations as the result of dissolution precipitation or localised conversion. The scale bar is 100 nm. Adapted with permission from ref. 51. Copyright 2017 Springer Nature.

Yang *et al*. demonstrated two methods to localise nanoparticles by spatial regulation using the sacrificial-template synthetic method, combining ZnO nanorods with AuNPs, forming ZnO@AuNPs before the addition of the linker to form Au–ZIF-8 composites (Fig. 10).^51^ The location of the nanoparticles was adjusted by controlling the MOF's crystallisation behaviour, which was dependent on the linker concentration. At high linker concentrations, nanoparticles were located near the oxide (Zn source), following the ‘dissolution precipitation mechanism’. In this process, a high concentration of 2-methylimidazole (Hmim) was used, which coordinated to zinc ions from the oxide, forming Zn(Hmim)_4_^2+^. At this concentration, nucleation deposits onto the surface of the oxide, leading to the growth of ZIF-8 around the nanoparticles. In contrast, a low linker concentration triggered the ‘localised conversion mechanism’, immobilising nanoparticles closer to the MOF surface due to insufficient formation of Zn(Hmim)_4_^2+^ for the growth of ZIF-8. As MOF nucleation occurs close to the surface of the oxide, ZIF-8 formation pushes the nanoparticles outwards. The feasibility of this particular NP-MOF composite synthesis was applicable for other oxides, including ZnO, Al_2_O_3_, and Cu_2_O, to obtain ZIF-8, MIL-53, and HKUST-1.

**Fig. 11:**
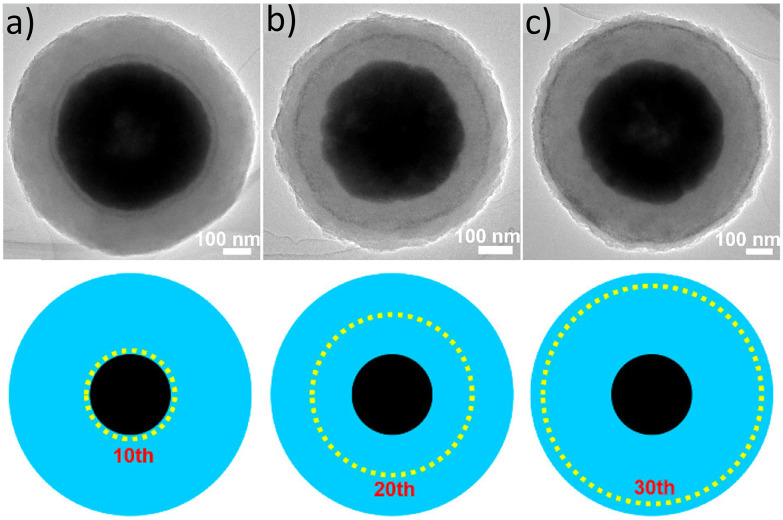
TEM images of (a) Fe_3_O_4_@MIL-100(Fe)–Pt(10), (b) Fe_3_O_4_@MIL-100(Fe)–Pt(20), and (c) Fe_3_O_4_@MIL-100(Fe)–Pt(30) with schematic representation below each image. Adapted with permission from ref. 52. Copyright 2020 American Chemical Society.

In other works, Liu *et al*. designed a Fe_3_O_4_@MIL-100(Fe)–Pt catalyst to reduce 4-nitrophenol by controlling the deposition of PtNPs onto different layers of MIL-100 surfaces during the growth process (Fig. 11).^52^ The MIL-100(Fe) shell thickness of 150 nm (40 layers) surrounding the Fe_3_O_4_ core remained uniform across all samples, and Pt loading increased from 1.37, 1.52, and 1.84 wt% as PtNPs were deposited at the 10^th^, 20^th^, and 30^th^ layers, respectively. This increase in loading was due to the higher available surface area of MIL-100 for PtNP deposition. The difference in PtNP placement led to different reactivities of the catalyst due to the diffusion distance of 4-nitrophenol towards the catalyst (see section 5).

### 2.3. One-pot synthesis approach

The last approach is the “one-pot synthesis” method, which involves mixing all reactants of the MOF and nanoparticles into a single reactor. This approach is more compatible with scaling up synthesis, reducing production costs and time. However, the rate of nanoparticle formation and MOF growth needs to be precisely controlled to achieve the desired localisation of nanoparticles in the MOF.^53^

**Fig. 12:**
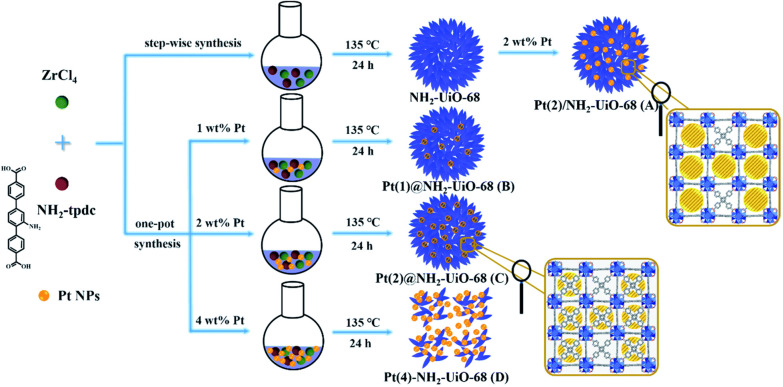
Schematic diagram of the two synthesis routes described by Guo *et al*.: step-wise synthesis of Pt/NH_2_–UiO-68 and one-pot synthesis of Pt@NH_2_–UiO-68. Reprinted with permission from ref. 54. Copyright 2019 Royal Society of Chemistry.

Guo *et al*. compared two synthetic methods to form NH_2_–UiO-66 composites with PtNPs: one-pot synthesis and step-wise synthesis, yielding Pt@NH_2_–UiO-68 and Pt/NH_2_–UiO-68, respectively (Fig. 12).^54^ These two methods resulted in different spatial localisation of PtNPs; the one-pot method yielded a flower-like MOF structure with PtNPs dispersed throughout the MOF petals instead of inside the pores due to the larger nanoparticle size (4 nm). The stepwise method mixed pre-synthesised PtNPs with NH_2_–UiO-68, resulting in PtNPs localised on the MOF's external surface. The electron mapping analysis revealed that PtNPs of Pt/NH_2_–UiO-68, located closer to the MOF surface, were more visible than PtNPs embedded inside Pt@NH_2_–UiO-68. The location of the PtNPs was further validated by XPS due to the resolution of the technique, with a higher intensity of Pt 4f peaks for Pt/NH_2_–UiO-68 compared to Pt@NH_2_–UiO-68. This difference in location signifies that PtNPs are located at different surface defect states in the hybrid structure, affecting the photocatalytic activity (see section 5).

It is also possible to utilise the one-pot solvothermal method for the time-saving synthesis of PtCo@UiO-66–NH_2_, which can be used in cinnamaldehyde hydrogenation reactions.^55^ In one solution, the hexachloroplatinic acid hexahydrate (H_2_PtCl_6_·6H_2_O, Pt precursor) and 2-aminoterephthalic acid (BDC–NH_2_, MOF linker) were mixed, while another solution containing cobalt oxide (CoO, Co precursor) and zirconium tetrachloride (ZrCl_4_) was prepared. Under solvothermal conditions, the precipitate was formed and isolated, then subsequently reduced using a 20 vol% H_2_/N_2_ flow at 200 °C for 4 h. Due to the presence of Pt and Co metal precursors and acetic acid, CoO was fully dissolved, resulting in no Co or CoO nanoparticle formation. This condition fully exhausted the Co supply, yielding mainly PtCo alloy particles. However, TEM images showed that this strategy could not prevent Pt or PtCo alloy nanoparticles from forming on the external MOF surface.

A facile one-pot synthesis for Pd@MOF without the use of an additional stabilising agent is also possible, and this concept was introduced in 2014.^56^ Instead of the typical 1,1′-biphenyl-4,4′-dicarboxylic acid (bpdc) used in UiO-67, 2,2′-bipyridine-5,5′ dicarboxylate (bpydc) was selected for the strong coordination of the pyridyl moiety to Pd. In this one-pot reaction, the formation of nanoparticles and MOFs co-occurred. Once PdNPs were formed, they were stabilised by the bpydc ligands, allowing ZrCl_4_ to assemble with the ligands on the nanoparticle surface, forming UiO-67. This interaction prevented aggregation, ensuring a homogenous distribution of encapsulated PdNPs.^56^

In a more recent study, a facile, *in situ* capping/reducing agent-free one-pot synthesis of 10%Pt@MOF-T3 was developed.^53^ The synthesis involved stirring ZrOCl_2_·8H_2_O, bpdc, Pt(ii)–bipyridine complex (Pt(ii)(Ph)_2_–bpydc), and benzoic acid in DMF at 120 °C for 24 h. PXRD analysis revealed that increasing the amount of Pt(ii) complex disrupted the MOF's crystallinity due to the steric hindrance of the complex. While increasing the number of Pt(ii) complexes destroyed the integrity of the MOF, an increase in the synthesis temperature helped the formation and encapsulation of PtNPs in the MOFs, ultimately resulting in larger and more crystalline MOF composites with well-distributed PtNPs, confirmed by XPS and TEM analysis. For this capping/reducing agent-free synthesis, all reactants played a role in the NP-MOF composite formation; with DMF exhibiting a reducing ability, which was inferred by XPS analysis revealing only peaks characteristic of Pt^2+^ when DMF was replaced by DMSO.

**Fig. 13:**
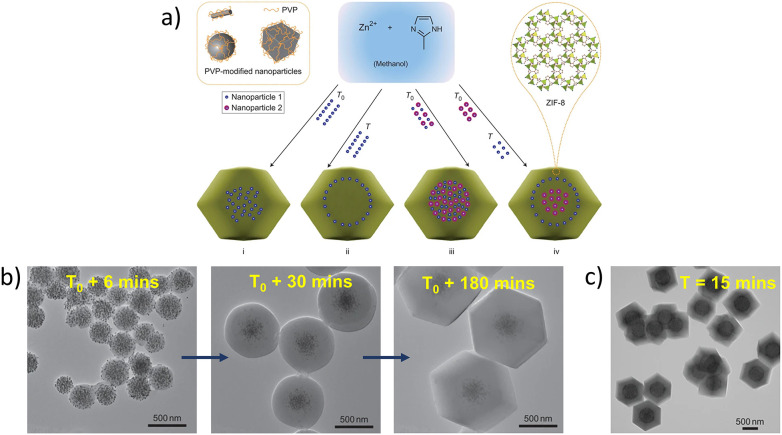
(a) A diagram showing how the addition sequence of the PVP-modified nanoparticles during MOF synthesis played a role in the spatial distribution of the incorporated PVP-modified nanoparticles within the ZIF-8 crystals at *T*_0_ or *T* during the MOF synthesis, (b) TEM images of the intermediate products of Au nanoparticle/ZIF-8 hybrid crystals collected at different reaction times: 6 minutes, 30 minutes, and 180 minutes after nanoparticle addition, (c) TEM image of hybrid crystals obtained when Au nanoparticles AuNPs were added 15 minutes after the initiation of the reaction. Adapted with permission from ref. 57. Copyright 2012 Springer Nature.

The spatially distributed encapsulation of different ligand-functionalised or unmodified nanoparticles in ZIF-8 can be achieved using one-pot synthesis by controlling the addition time of nanoparticles, either at the beginning (*T*_0_) or after a specified amount of time (*T*) (Fig. 13a).^57^ Four types of spatial distributions were reported, where the composite formation *via* Type I and Type II is shown in Fig. 13b and c. The MOF formation was monitored by TEM, showing the encapsulation of 13 nm AuNPs within a thin layer of ZIF-8 resembling ‘hybrid spheres’ in the first six minutes of the reaction, followed by the growth of NP-free shells surrounding the hybrid sphere after 30 minutes and 180 minutes (Fig. 13b). UV-visible spectroscopy confirmed nanoparticle encapsulation indicated by the red shift of the AuNP plasmon resonance band from 520 to 540 nm and increased spectral intensities, suggesting hybrid sphere growth. Amphiphilic PVP played a crucial role in stabilising PVP–AuNPs on the ZIF-8 surface through weak interactions between coordination-polymer spheres and zinc atoms. However, excessive use of PVP led to competition between free PVP and PVP–AuNPs, whereby PVP–AuNPs can no longer adsorb on ZIF-8, causing the MOF to stop growing. This method was also applied to other nanoparticles, such as Pt, CdTe, Fe_3_O_4_, and Ag cubes, demonstrating the versatility of nanoparticle encapsulation by altering the MOF growth sequence.

### 2.4. Other approaches

**Fig. 14:**
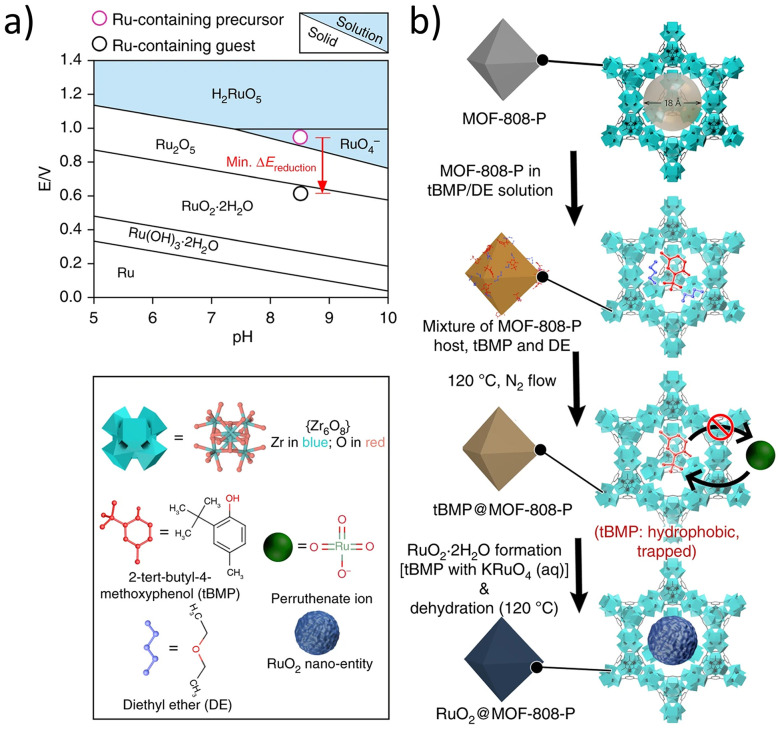
(a) The Pourbaix diagram for the Ru–H_2_O system, (b) schematic diagram of the Pourbaix-enabled guest synthesis (PEGS) strategy for RuO_2_@MOF-808-P. Reprinted with permission from ref. 58. Copyright 2019 Springer Nature.

In terms of more auxiliary methods of synthesis, in 2019, Wang *et al*. proposed the use of the Pourbaix-enabled guest synthesis (PEGS), benefiting from the Pourbaix diagram to predict the condition when the metal is in the metallic or ionic state (Fig. 14a).^58^ They claimed that this method solves the challenge of including guest particles inside MOFs using the conventional “ship-in-bottle” route. PEGS required an optimised condition in terms of the difference in redox potential between the reactants and the pH of the solutions. 2-*tert*-butyl-4-methylphenol (*t*BMP) lipid and diethyl ether were introduced into MOF-808, making a suitable hydrophobic environment for the entrapment of potassium perruthenate (KRuO_4_). Using temperature-controlled selective-desorption conditions, *t*BMP and diethyl ether were desorbed from the external surface, hence directing RuO_2_ nanoparticles inside the MOF (Fig. 14b).

In 2020, Su *et al*. reported a surfactant and reductant-free synthesis for Ag, Au, and PdNPs in Zr-based MOFs. This method relied on the redox activity of the MOF by mixing the tetrathiafulvalene (TTF)-containing MOF with methanolic solutions of metal salts (AgNO_3_, PdCl_2_, or KAuCl_4_).^59^ The successful formation of nanoparticles was confirmed by a colour change from orange to green, with the nanoparticles *ca*. 1.6 nm in size and located mostly inside the smaller MOF pores, as determined by single-crystal synchrotron X-ray diffraction. As this method relied solely on the redox ability of MOFs, not all metal precursors were fully reduced, which was confirmed by XPS, showing the presence of Pd and Au as a mixture of Pd^0^/Pd^2+^ and Au^0^/Au^3+^ states. This study demonstrated the use of redox-active MOFs for the *in situ* generation and stabilisation of ultra-small noble metal nanoparticles, which selectively immobilise nanoparticles within the small cavity, leaving the large mesopores open for other applications. A similar approach was adapted by Qiu *et al*. in 2022 to synthesise AgNPs@Zr–TTFTB as a photocatalyst for the degradation of sulfamethoxazole (SMZ).^60^

Physical ultrasonic mixing is another simple method that can be utilised in MOF-NP composite synthesis, as shown by Zhang *et al*., to decorate ultrathin Zr–TCPP (TCPP = tetrakis(4-carboxyphenyl)porphyrin) or Zr–TCPP(Pd).^61^ The PVP-stabilised PtNPs were successfully deposited on the nanosheet. ICP-OES and EDS analysis confirmed that the experimental Pt content coincides with the theoretical value of 2%.

Additionally, Chai *et al*. demonstrated AuNP synthesis *via* electrodeposition onto UiO-66-coated carbon cloth (CC) substrate.^62^ The UiO-66 coating was achieved by the conventional solvothermal method on a plasma-cleaned CC substrate. The substrate was immersed in H[AuCl_4_] aqueous solution as gold precursor in a three-electrode cell, then a periodic galvanostatic pulse current with different numbers of cycles was run for electrodeposition. SEM images show no visible change after electrodeposition, but the EDS analysis revealed a uniform distribution of gold, meaning that the AuNPs are encapsulated in the UiO-66/CC film. Though AuNPs were encapsulated, they acted as the catalytic centre for H_2_O_2_ electroreduction.

Sadakiyo *et al*. utilised the arc plasma deposition method and succeeded with the gram-scale preparation of M/MOFs (M = metal nanoparticles), and for the first time, the formation of a Pt–Co nanoalloy using synchronous shots of dual arc plasma guns.^63^ MOF powder was irradiated with arc plasma shots of the desired metal elements with the frequency range of 1–2 Hz at the applied voltage of *ca.* 140 V, yielding Pt/ZIF-8 (0.82 wt% metal loading), Pd/ZIF-8 (0.79 wt%), Ru/ZIF-8 (0.93 wt%), Ru/MIL-101 (1.09 wt%), Pd/UiO-66–NH_2_, (1.10 wt%), and Pd–PVP/UiO-66–NH_2_ (0.83 wt%, PVP = polyvinylpyrrolidone). PXRD and scanning TEM (STEM) analysis confirmed that MOF structures were retained for all samples, and nanoparticles around 2 nm were located on the external surface rather than homogenously distributed within MOF pores. With the small counts of plasma shots, small nanoparticles were deposited onto the surface of the MOF, which in turn grew as more plasma shots were applied. With a further increase in the shot counts, nanoparticles wedded together to form a rod shape instead of growing larger.^63^ Overall, the ADP method is feasible for large-scale synthesis and applicable to various MOFs and metal elements. The particle size is independent of the type of MOF, metal source, or voltage, but rather on the amount of shots applied.

Overall, the diversity of NP-MOF synthesis is not limited to the methods reported in this review (Table 1). It is important to take into consideration the nature of the parent MOF, such as pore size and physical/chemical stability, to match the appropriate NP-MOF formation with the desired nanoparticles’ size and location. Furthermore, modification to the MOF structure could further enhance the incarceration of nanoparticles, reported examples are discussed in the next section.

**Table 1:** Summary of the advantages and challenges of the synthesis methods discussed in section 2

Method	Advantage	Limitations
Ship-in-bottle	• Leverage MOF porosity to limit NP growth	• Multi-step reaction
	• DSM – high selectivity for interior NP growth	• Partial NP reduction is possible
	• MOF acts as stabilising agent for the NPs	• Difficult to realise precise control of NP loading and location
		• NP growth could lead to the destruction of MOF pores or its integrity
Bottle-around-ship	• Spatial control over NP location	• Require stabilised nanoparticles prior to MOF formation
	• Encapsulation-structure composite	• Template may be needed
		• Large interfacial energy barrier between two different materials
One-pot	• Scalable synthesis	• Need to fine-tune the NP-MOF formation rate
	• Fast synthesis	• Less structure control

## 3. Ligand-functionalised MOF-assisted nanoparticle stabilisation and growth

The success of forming NP@MOF composites relies on several factors, such as the type of metal precursors, pore size of the MOF, the nanoparticle nucleation process, and how effectively the host MOF can stabilise the metal nanoparticles. Typically, functional groups on the MOF linker or metal clusters serve as binding sites for hosted metal nanoparticles based on hard–soft acid–base (HSAB) interactions. A method for modifying MOF scaffolds includes SALI to introduce the requisite binding sites. Subsequently, metal precursor impregnation, AIM, or SIM are techniques used to incorporate the nanoparticles.

SALI is a post-synthetic modification technique that is often implemented for NU-1000, typically introducing a carboxylic acid ligand to the Zr_6_ node, which can directly coordinate to Zr or *via* an acid–base reaction with terminal OH or OH_2_ groups at the Zr_6_ nodes.^64^ After the first report in 2013 on NU-1000 SALI functionalisation with perfluoroalkyl carboxylic acids in the MOFs,^64^ similar SALI-based modifications have since been demonstrated, such as functionalisation with a fluorenylmethyloxycarbonyl (Fmoc)-protected triglycine peptide and 2,6-diacetylaminopyridine moiety,^65^ several substituted benzoates,^13^ and various carboxylic functional groups (CFGs).^66^ Subsequently, the functionalised NU-1000 was able to host nanoparticles. For example, Huang *et al*. demonstrated the use of perfluoroalkane-functionalised NU-1000 decorated with PdNPs as a catalyst for the direct C–H arylation of indoles in water.^67^ The perfluoroalkane chains themselves had been reported as stabilisers for palladium (Pd), ruthenium, and silver nanoparticles, with the extent of the chains giving rise to a hydrophobic environment within the MOF pores. This enhanced the adsorption of organic reactants in aqueous solution, accelerating the catalytic reaction.^67–70^ Similarly, NU-1000 modified with 4-carboxy-phenylacetylene (PA) *via* SALI was used to anchor AuNPs for the catalytic reduction of 4-nitrophenol into 4-aminophenol.^12^

**Fig. 15:**
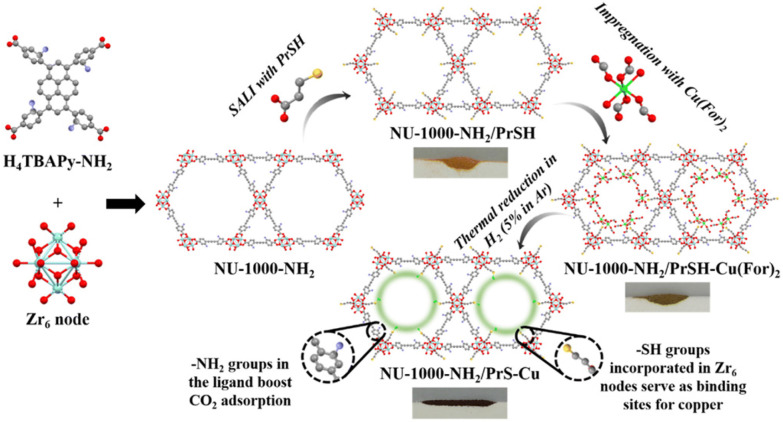
Schematic representation of the synthesis procedure to produce NU-1000–NH_2_/PrSH–Cu, highlighting the reasoning behind the installed functionalities and the difference in colour of the powders during the synthesis steps. Reprinted with permission from ref. 24. Copyright 2024 Royal Society of Chemistry.

Many studies have employed different types of functional groups for MOF modification, with N-rich or S-rich moieties being the most common, especially for nanoparticles such as Au, Ag, Pd, and Pt. In a recent study, NU-1000 was modified with amino (–NH_2_) and thiol (–SH) groups to improve CO_2_ adsorption and conversion performance (Fig. 15).^24^ NU-1000 typically has low CO_2_ affinity; however, with the –NH_2_ functionalisation, the resulting MOF showed enhanced CO_2_ adsorption compared to pristine NU-1000. Further modification with a thiol precursor produced NU-1000–NH_2_/PrSH, where the thiol groups served as the binding sites for CuNPs due to their complementary ‘soft’ characteristics. XPS confirmed the presence of the S–Cu interaction, and EDS line scanning showed a homogeneous distribution of Cu. The thiol group also prevented the migration of Cu atoms from the cavities to the outer surface, thereby reducing nanoparticle aggregation.

**Fig. 16:**
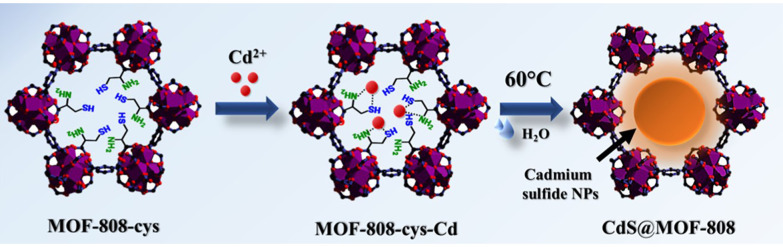
Synthesis scheme of CdS@MOF-808 composites produced by post-synthetic ligand exchange (PSLE) of MOF-808 with l-cysteine (l-cys), followed by the growth of CdSNPs after temperature treatment. Reprinted with permission from ref. 7. Copyright 2022 American Chemical Society.

Thiol group functionalisation is also prevalent with other metals such as: Au, Ag, and Pd due to the soft–soft acid–base interactions.^71^ Examples of thiol-functionalised MOFs include Ag@Zr–DMBD for the CO_2_ cycloaddition reaction^72^ and Ag@UiO-66–SH for the A^3^ coupling reaction.^73^ Ghosh *et al*. demonstrated an interesting approach using metal sulfide nanoparticles with MOFs to create a new photocatalyst for HER (Fig. 16).^7^ In this study, the post-synthetic ligand exchange (PSLE) of formate ions within MOF-808 with l-cysteine (l-cys), followed by the growth of cadmium sulfide (CdS) nanoparticles, yielded MOF-808–cys–CdS. Characterisation methods such as ^1^H-NMR (nuclear magnetic resonance), FT-IR (Fourier transform infrared spectroscopy), and elemental analysis confirmed successful ligand exchange on the Zr clusters. l-cys was chosen specifically due to the favourable interactions between the ‘soft’ sulfide and ‘soft’ Lewis acid, Cd, and the ability of the NH_2_ of l-cys to replace formate within the Zr cluster. In addition to Cd^2+^, other metals such as Pd^2+^ and Hg^2+^ were also studied with the l-cys-functionalised MOF-808, where the functionalised MOF-808 showed heavy metal ion trapping with a greater than 99% removal rate.^7^

Borah *et al*. also recently reported the use of thiol-rich Zr–DMBD MOF (DMBD = 2,5-dimercapto-1,4-benzenedicarboxylate) for incarcerating bismuth nanoparticles (BiNPs) as electro- and photocatalysts.^74^ BiNP@Zr–DMBD was obtained through the typical “ship-in-bottle” route using Bi(NO_3_)_3_·5H_2_O as a bismuth source and reduced by NaBH_4_. The thiol group acted as the binding site for the bismuth precursor and controlled the growth of BiNPs as the Bi–S interaction prevented intermolecular interaction and polymerisation of Bi^3+^. The obtained BiNPs were around 4 nm and found to be distributed only inside the MOF matrix as determined by TEM analysis.

**Fig. 17:**
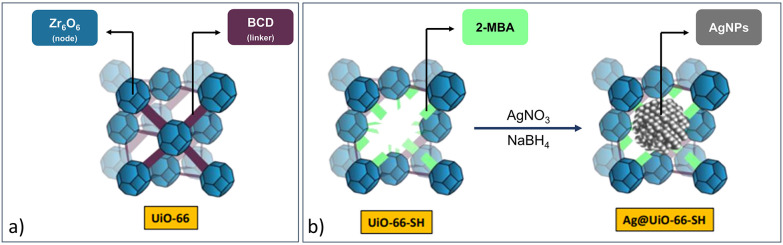
(a) A diagram of UiO-66 and (b) schematic representation for the synthesis of Ag@UiO-66–SH composite using defective UiO-66–SH with AgNO_3_ as the silver source, then reduced with NaBH_4_. Adapted with permission from ref. 73. Copyright 2022 American Chemical Society.

As an alternative to the traditional impregnation of intact MOFs, Aparna *et al*. used defective UiO-66 to host AgNPs.^73^ In this study, defect sites with thiol functionalities served as anchoring points for AgNPs. The synthesis of UiO-66–SH included an excess of 2-mercaptobenzoic acid (2-MBA) as a modulator, by replacement of the benzene dicarboxylic acid (BDC) linker (Fig. 17). The successful linker exchange was demonstrated by ^1^H NMR with 43% replacement of BDC by 2-MBA. AgNPs were introduced *via* silver nitrate (AgNO_3_) impregnation with coordination to the thiol of 2-MBA before reduction with sodium borohydride (NaBH_4_). ICP-MS analysis showed a low Ag content of 17.29 ppm, and Brunauer–Emmett–Teller (BET) analysis determined that some of the AgNPs were located in the MOF pores. The resulting Ag@UiO-66–SH exhibited moderate to excellent catalytic activity for a three-component A^3^ coupling reaction. The importance of the thiol functionalisation was demonstrated with 95% conversion despite the low Ag catalyst loading of only 0.3 mol% compared to UiO-66 stabilised AgNPs (25% conversion). Therefore, the catalytically active species in this study were the AgNPs on thiolated MOF support, although the authors did not extend this discussion.^73^

Amine-functionalised UiO-66 is used widely as a host for catalytically active nanoparticles. Qiu *et al*. used UiO-66 and UiO-66–NH_2_ as hosts for AuNPs and PtNPs in photocatalytic applications.^75^ XPS showed that the electron-withdrawing amine group influences the electron binding energy of both Au and Pt. This amine-mediated enhancement also increased the photocatalytic activity by promoting electron transfer. Electrochemical impedance spectroscopy (EIS) demonstrated that the Au or Pt loading, in addition to the amine functionalisation, resulted in the faster interfacial transfer of electrons and electron–hole separation, improving the photocatalytic activity of the catalysts. An interesting observation from HR-TEM showed that Pt(200) planes were more common in Pt/UiO-66–NH_2_, whereas more Pt(111) planes were observed in MIL-125–NH_2_. Furthermore, samples with Pt(200) or Pt(111) possessed contrasting catalytic activity; Pt(200) in Pt/UiO-66–NH_2_ had a suppressing effect on the oxidation of benzoyl alcohol, while Pt(111) in MIL-125–NH_2_ exhibited an increase in activity. The interference of the metal plane was not observed when Au/UiO-66–NH_2_ was used for the same catalytic reaction.

Additionally, the amine group can also serve as an anchor for other organic compounds, *e.g.* thiol groups. Patra *et al*. reported the advantage of post-synthetic modification (PSM) of thiol-containing groups over direct synthesis with thiol ligands for the preparation of Ag@UiO-66–NH–SH, which was investigated for CO_2_ fixation.^76^ PSM overcomes challenges such as multistep synthesis, high-temperature requirements, and instability. In this case, PSM was achieved by reacting UiO-66–NH_2_ with sulfurising agents such as thioglycolic acid or 3-mercaptopropionic acid. The thiol group effectively anchored AgNPs, which was confirmed by the reduction of the thiol peak intensity in FTIR and a shift of the thiol peak in XPS, indicating S–Ag interaction.^76^

**Fig. 18:**
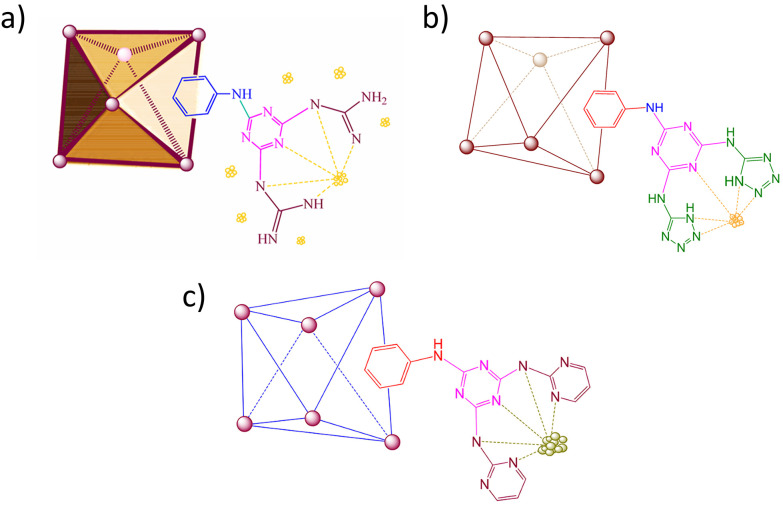
Simplified structures of (a) UiO-66–NH_2_@cyanuric chloride@guanidine/PdNPs,^77^ (b) UiO-66–NH_2_@cyanuric chloride@5-amino tetrazole/AuNPs,^78^ and (c) UiO-66–NH_2_@cyanuric chloride@2-aminopyrimidine/PdNPs.^79^ Adapted with permission from ref. 77 and 79. Copyright 2023 American Chemical Society. Adapted with permission from ref. 78. Copyright 2023 Springer Nature.

A multi-step PSM of UiO-66–NH_2_ with cyanuric chloride and guanidine was employed to synthesise UiO-66–NH_2_@cyanuric chloride@guanidine as a catalyst for C–C coupling reactions.^77^ Subsequent metalation with palladium acetate (Pd(OAc)_2_) and reduction with hydrazine hydrate resulted in the formation of PdNPs (46–47 nm) on the external surface of the MOF crystals (Fig. 18a). The large PdNPs resulted from the use of hydrazine hydrate as a weak chemical reductant, causing localisation on the outside of the MOF; however, despite this, TEM showed well-dispersed nanoparticles. This PSM strategy helped to stabilise the PdNPs, resulting in enhanced catalytic activity due to guanidine's synergistic effect and an improvement in the intrinsic stability of UiO-66–NH_2_ in an aqueous environment for recycling. Similarly, UiO-66–NH_2_@cyanuric chloride@5-amino tetrazole/AuNPs (Fig. 18b) and UiO-66–NH_2_@cyanuric chloride@2-aminopyrimidine/PdNPs (Fig. 18c) were synthesised for A^3^-coupling reactions.^78,79^ The presence of N-rich groups enhanced the electronic structure and chemical stability of the nanoparticles through coordination.

Furthermore, a recent study by Zhang *et al*. reported the preparation of a yolk shell photocatalyst containing nickel phosphide (Ni_2_P) NPs and dicopper oxide (Cu_2_O) nanoparticles for oxidative coupling of amine.^80^ Ni_2_P NPs were encapsulated within OH–NH_2_–UiO-66 through an *in situ* synthesis method. Subsequent photodeposition of copper sulfate pentahydrate solution using a Xe lamp yielded Ni_2_P@OH–NH_2_–UiO-66@Cu_2_O. The resulting structure allows for the embedded Ni_2_P and surface Cu_2_O NPs to separately act as the electron and hole collectors, respectively. The significance of modifying the parent MOF structure by partial replacement of 2,5-dihydroxyterephthalic acid with 2-aminoterephthalic acid was reflected in the increased light absorption compared to the parent NH_2_–UiO-66. Overall, the addition of hydroxy groups resulted in defective sites where the light adsorption was further enhanced by the nanoparticles.

Variation of the functional group of UiO-66 (–H, –F, –NH_2_, and –OH) has been shown to result in different catalytic efficiencies for the hydrogenation of alkynes.^81^ The Pt encapsulation used the electronic attraction of the Pt complex with unsaturated coordinated Zr atoms in UiO-66. By using an acidic condition, the protonated Zr–O sites are capable of adsorbing more Pt complexes due to the electronic attraction, whereas in a basic condition, an electronic repulsion is experienced. Even though PtNPs formed *via* H_2_/Ar reduction were similar, the electron density of PtNPs was different due to the electron acceptor/donor nature of the functionalised group. As –F is an electron acceptor, this renders the PtNPs’ electron density low, as well as increasing the catalyst's hydrophobicity, therefore improving the catalytic activity in the order of F > H > NH_2_ > OH. The study by Feng *et al*. is also in agreement that the amine functionalisation can provide additional electrostatic interactions when the MOF is in aqueous solution (UiO-66–NH_3_^+^) with the dissociated metal precursor, H_2_PdCl_4_, into H^+^ and PdCl_4_^2−^.^82^

**Fig. 19:**
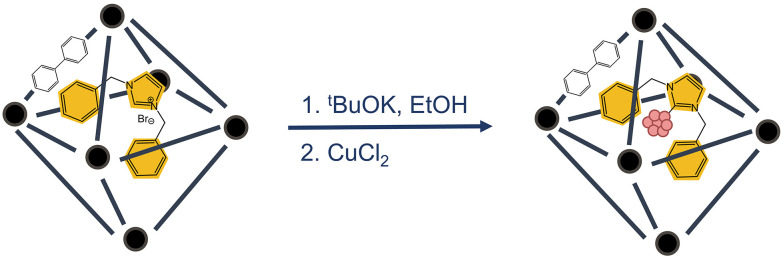
Synthesis reaction scheme to produce 2Bn–Cu@UiO-67 from UiO-67–NHC by addition of base and copper salt. Adapted with permission from ref. 88. Copyright 2022 John Wiley and Sons.

N-heterocyclic carbenes (NHC) have emerged as versatile stabilising agents for metal nanoparticles due to their strong σ–donating properties for coordinating metal surfaces.^83–85^ Some examples include PdNPs@NHC@ZIF-8,^86^ or Pd–NHC–MIL-101(Cr).^87^ Wang and coworkers reported the ability to embed NHC-ligated Cu single atom sites in UiO-67–NHC, which could catalyse methane electrosynthesis from CO_2_ (Fig. 19).^88^ After embedding the NHC within the pores of UiO-67, treatment under basic conditions induced deprotonation at the carbene carbon, which was subsequently followed by facile coordination of Cu to the NHC, ultimately yielding 2Bn–Cu@UiO-67 containing the NHC–Cu complex. The catalytic performance of 2Bn–Cu@UiO-67 was evaluated against a control sample prepared without alkali activation and Cu coordination (2Bn–ACu@UiO-67). The superior activity of 2Bn–Cu@UiO-67 was attributed to the σ-coordination of NHC ligands, which increased the charge density on the MOF surface. This enhancement facilitated electrophilic binding and promoted selective methane formation during CO_2_ reduction. This study highlights the broader applicability of MOF-hosted single-atom catalysts.

In 2025, a hierarchically porous material for photo-thermal CO_2_ cycloaddition was developed by Jiang *et al*.^89^ The wet impregnation of ionic liquid, [BmIm][AuCl_4_] (BmIm = 1-methyl-3-butylimidazolium), into UiO-66, followed by *in situ* pyrolysis under a H_2_/Ar flow, yielded Au@H–UiO-66. The Au@H–UiO-66 composite retained its structure with AuNPs dispersed within UiO-66. N_2_ adsorption–desorption isotherms and TEM confirmed the presence of mesopores, resulting in an enhancement of mass transfer, hence an 11.5-fold increase in chloropropene carbonate production to 92% yield when compared to the parent UiO-66. This work provided evidence that the [Bmim][AuCl_4_] can serve as both the AuNP precursor and the mesopore-forming agent.^89^

## 4. Location identification methods for nanoparticles in NP-MOF composites

Increasingly advanced scientific methods have become accessible for detecting and precisely locating metal nanoparticles on/within MOFs. Various techniques are already employed in research for the characterisation of the NP-MOF composites, including NMR, PXRD, FT-IR, SEM, TEM, BET analysis, thermal gravimetric analysis (TGA), ICP-MS, energy-dispersive X-ray (EDX), and elemental mapping analysis. A thorough understanding of both qualitative (*e.g.*, particle size or morphology) and quantitative (*e.g.*, percentage of metal nanoparticle loading) aspects of nanoparticles allows for better prediction of the catalytic activity of the composite.

While TEM can be used to visualise the distribution pattern of nanoparticles within the MOF structure, it does not accurately distinguish between nanoparticles situated within the MOF crystals or on their external surfaces. The reduction of N_2_ adsorption indicates pore occupancy either by the PSM ligands or nanoparticles, but this method could still be misleading if nanoparticles merely block the pore entrances. Alternatively, PXRD is another common characterisation technique used to determine MOF crystallinity, showing that it is possible to observe characteristic patterns from the incorporated nanoparticles when they are large enough. However, when such peaks are absent, it is generally inferred that the nanoparticles are either too small for detection or present at too low a concentration. Due to these limitations, it is essential to include higher precision techniques to assist in locating nanoparticles within MOFs.

### 4.1. Microscopic techniques

**Fig. 20:**
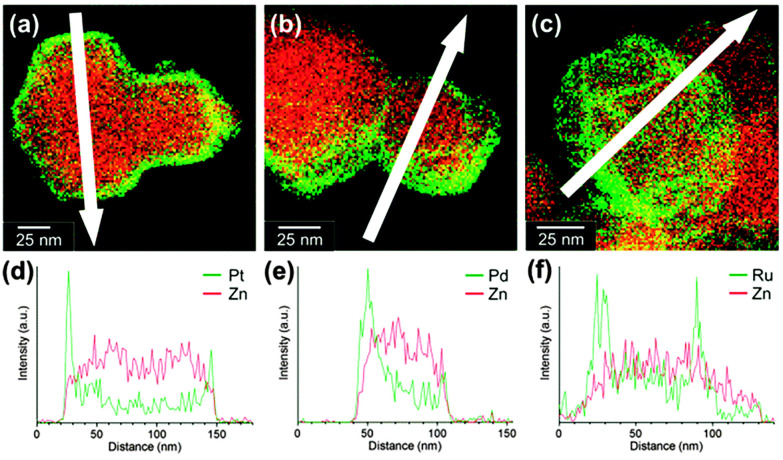
STEM-EDS maps of (a) Pt/ZIF-8, (b) Pd/ZIF-8, and (c) Ru/ZIF-8. Line profiles of (d) Pt/ZIF-8, (e) Pd/ZIF-8, and (f) Ru/ZIF-8 along white arrows (green: Pt–M, Pd–L, or Ru–L; red: Zn–K). Reprinted with permission from ref. 63. Copyright 2016 Royal Society of Chemistry.

**Fig. 21:**
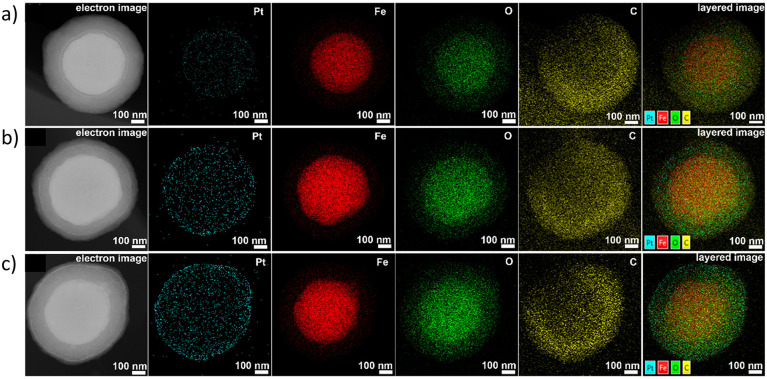
Electron image, elemental mapping images, and EDX layered images of (a) Fe_3_O_4_@MIL-100(Fe)–Pt(10), (b) Fe_3_O_4_@MIL-100(Fe)–Pt(20), and (c) Fe_3_O_4_@MIL-100(Fe)–Pt(30). Adapted with permission from ref. 52. Copyright 2020 American Chemical Society.

In many studies involving metal nanoparticles, microscopic techniques such as TEM, STEM, or HAAD-STEM combined with EDS are essential tools to determine nanoparticle size and distribution. STEM-EDS line scans determined the core–shell structures of ZIF-8 containing Pt, Pd, and RuNPs, showing strong signals from nanoparticles mainly observed on the edges of MOFs (Fig. 20, section 2.1).^63^ In another study, electron and EDX images of Fe_3_O_4_@MIL-100(Fe)–Pt composites were used to study the location of PtNPs at different MIL-100 layers, as shown in Fig. 21 (section 2.2).^52^ In the electron images, the PtNPs were identified by their higher atomic mass than the MOF components, hence bright white rings. Additionally, EDX confirmed that the signals of PtNPs became more pronounced when they were located closer to the MOF's surface (Fig. 21, second column). The locational difference of PtNPs was reflected in the catalytic ability of Fe_3_O_4_@MIL-100(Fe)–Pt, revealing that PtNPs at the 30^th^ layer, Fe_3_O_4_@MIL-100(Fe)–Pt(30), exhibited the highest activity due to the proximity of the PtNPs to the MOF surface (see section 5).^52^

**Fig. 22:**
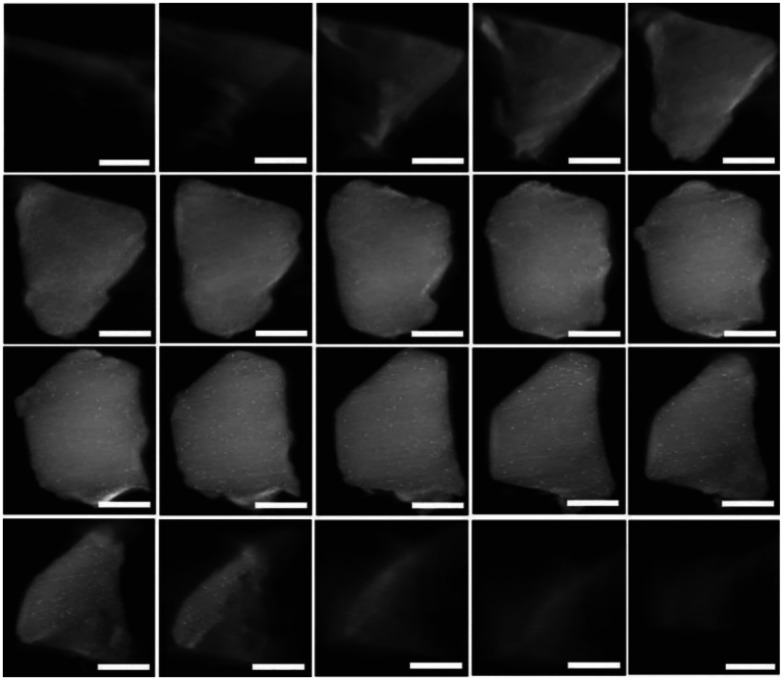
Reconstructed HAADF-STEM images of PtCuNPs throughout the Pt_1_Cu_2_@MIL-101 skeleton (observed from left to right and top to bottom). The scale bar on the images is roughly ∼200 nm. Reprinted with permission from ref. 91. Copyright 2019 Springer Nature.

Traditional 2D microscopy images are limited in their ability to fully depict the 3D arrangement of NP-MOF composites. Therefore, electron tomography is gaining popularity as it captures images at various sample angles with constant tilt increments, enabling 3D reconstruction of nanoparticle distribution. For instance, the distribution of PdNPs in Pd@MIL-101 synthesised by DSM with a reductive atmosphere was observed using electron tomography, revealing approximately 1.8 nm PdNPs uniformly dispersed within pores of MIL-101 (section 2.1).^90^ Furthermore, this technique also showed the dispersion of AuNiNPs in AuNi@MIL-101 throughout the interior cavities of MOF (section 2.1).^32^ Similarly, Chen *et al*. used 3D construction of HAADF-STEM images at consecutive tilt angles from −62.6° to 62.6° with 2° tilt increments to locate PtCuNPs, which were found uniformly dispersed within the MIL-101 framework (Fig. 22; section 2.1).^91^

### 4.2. Different envelope density (DED) analysis

**Fig. 23:**
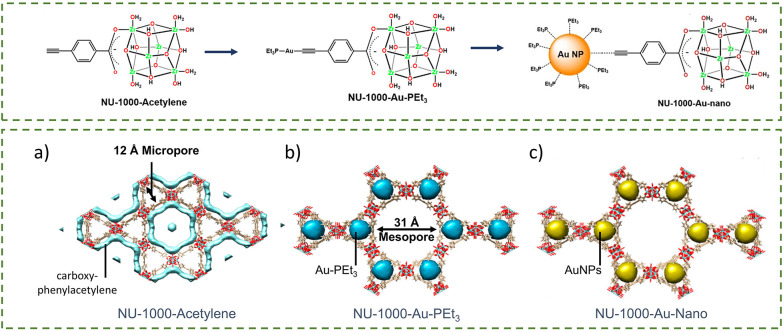
Different envelope density (DED) maps showing the location of (a) carboxy-phenylacetylene in NU-1000–acetylene, (b) Au–PEt_3_ in NU-1000–Au–PEt_3_, and (c) AuNPs in NU-1000–Au–nano. Adapted with permission from ref. 12. Copyright 2019 American Chemical Society.

Different envelope density (DED) analysis is another useful technique for tracking changes in electron density during reactions. DED analysis of synchrotron-derived X-ray diffraction data revealed the difference in electron density of carboxy-phenylacetylene in NU-1000–acetylene (Fig. 23a), electron density of Au–PEt_3_ in NU-1000–Au–PEt_3_ (Fig. 23b), which is the state before the formation of AuNPs in NU-1000–Au-nano (Fig. 23c). The shift of electron density from the mesopores to micropores was also evidenced in the decrease in the BET surface area.^12^

**Fig. 24:**
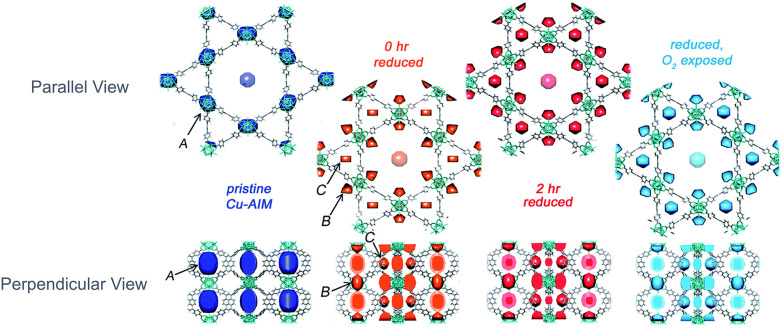
DED maps showing parallel (top) and perpendicular (bottom) views of *c*-axis for Cu–AIM in the pristine state (dark blue), at the beginning (red) and end of reduction at 200 °C (pink) and following re-oxidation (light blue). Adapted with permission from ref. 11. Copyright 2017 Royal Society of Chemistry.

In another study, Platero-Prats *et al*. used DED to follow the electron density change during the reduction of Cu-oxo species into metallic Cu(0) species.^11^ They defined three possible locations for Cu species as sites A, B, and C, as shown in Fig. 24. Using DED, the change in electron density was only observed after the formation of Cu(0). Initially, Cu-oxo clusters were located in the smaller cavity between Zr_6_ nodes (site A). The preference for the smaller cavity for metal precursor deposition rather than the larger hexagonal cavity has been observed previously using DED for NU-1000 treated with diethylzinc *via* ALD.^36^ Since MOF pores are the ideal location, the smaller pores tend to provide adsorption with the lowest adsorption energy and maximise the interaction; hence, site A has the highest electron density. Upon reducing the ALD-deposited Cu-oxo cluster, resulting in the appearance of CuNPs, electron density redistribution was observed towards site B and C, which are adjacent to the Zr_6_ node faces in the hexagonal channel and the triangular channel between the pyrene groups, respectively.^11^ After the reduction stage, copper existed in both nanoparticle and metal cluster forms, with the latter located favourably in site C. Then upon re-oxidation, the DED does not change significantly; there is a small decrease in electron density from site C. However, the authors stated that the electron density distribution observed with DED could not fully explain the formation of 4 nm CuNPs, which are larger in size than the NU-1000 pores, as well as the fact that the DED technique was not suitable for the analysis of materials on the external surface.^11^

### 4.3. PDF analysis

*In situ* synchrotron X-ray scattering experiments and PDF analysis have been used to monitor the growth of CuNPs.^92^ PDF analysis provides insights into interatomic distances, which can be used to monitor changes in short- and long-range bonding, crystal packing, and nanoparticle size. For example, PDF analysis of CuNPs@NU-901 and CuNPs@NU-907 revealed the appearance of new peaks in addition to those belonging to the pristine MOFs. For CuNPs@NU-901, new short and moderate-range peaks at ∼2.5 Å, 4.5 Å, 6.7 Å, 9.2 Å, and 13.4 Å were observed, while only peaks at ∼2.5 Å, 4.5 Å, and 6.7 Å were observed for CuNPs@NU-907. These new peaks were indicative of face-centred cubic packing of Cu metal, thus confirming the formation of CuNPs. The lower intensity of Cu peaks and the missing longer-range signals for CuNPs@NU-907 were attributed to smaller CuNPs in CuNPs@NU-907 compared to CuNPs@NU-901. Moreover, the particle size of CuNPs was calculated by subtracting the PDF of the composites from the PDF of pristine MOF, resulting in a differential PDF (dPDF). It was reported that the CuNPs in CuNPs@NU-901 and CuNPs@NU-907 were around 15–16 Å and 9–10 Å, respectively.

### 4.4. Hyperpolarised ^129^Xe NMR and positron annihilation

**Fig. 25:**
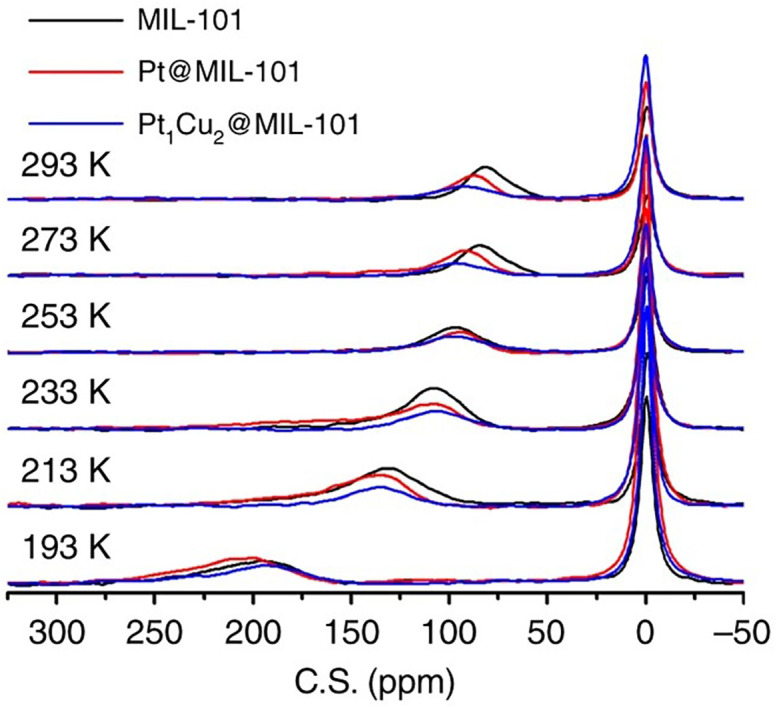
Temperature-dependent hyperpolarised ^129^Xe NMR spectra for MIL-101, Pt@MIL-101, and PtCu@MIL-101 at temperatures ranging from 193–293 K. Reprinted with permission from ref. 91. Copyright 2019 Springer Nature.

Hyperpolarised ^129^Xe NMR and positron annihilation (PA) are two additional techniques used to efficiently locate nanoparticles within MOFs, as seen by Chen *et al*. for PtNPs and PtCuNPs in MIL-101 (sections 2.1 and 4.1).^91^ Using solid samples, hyperpolarised ^129^Xe NMR tracks the interaction of Xe molecules, a non-invasive gas, within MOF pores, with Xe exhibiting a change in chemical shift from 0 ppm to more downfield values. This chemical shift indicates a change in pore size due to nanoparticle occupancy. Using temperature-variation ^129^Xe NMR analysis, lowering the temperature resulted in stronger Xe–Xe interactions, shifting peaks downfield for all samples (Fig. 25). When fixed at one temperature (293 K), the samples with the greatest peak shift were indicative of a smaller pore size, revealing that MIL-101 had the largest pore size, followed by Pt@MIL-101 and PtCu@MIL-101. This order was attributed to the larger size of the PtCuNPs compared to the PtNPs. The same result was observed when different amounts of PtCuNPs were loaded; the peaks were shifted downfield with higher loading content.^91^ Therefore, ^129^Xe NMR can provide quantitative information about nanoparticle loading within MOF pores; however, the technique is limited when the metal loading is very low, resulting in limited MOF occupancy.

Positron annihilation was also used in this work to further validate nanoparticle localisation inside the MOF pores due to the sensitivity of positrons towards disorders/defects/pores. The positron lifetime (*τ*) is the time difference between birth and annihilation of the positron; a longer lifetime correlates with larger pore sizes. From the experiment, three different lifetimes were reported (−*τ*1, *τ*2, and *τ*3), in which *τ*2 is the lifetime of the positron trapped by pores/defects in the MOF. In this work, *τ*2 was reported to have the highest intensity, indicating changes to the pores of MIL-101. When 10% PtCuNPs were loaded into the MOF, *τ*2 values decreased, indicating that the free space in the MOF became smaller, thus concluding that nanoparticles were located inside the pores.^91^

## 5. Effect of nanoparticles’ spatial localisation on catalytic activity

The spatial distribution of nanoparticles – within the MOF structure, near the external surface, or entirely outside – significantly impacts their catalytic activity. In porous MOFs, the structure may act as a molecular sieve, allowing reactants and products to diffuse. As a result, catalytic activity hinges on whether the reactants can access the active sites. Additionally, the interaction between nanoparticles and the MOF can modify the electronic structure, either enhancing or reducing the catalytic activity. The following reports are examples of the different spatial distributions of nanoparticles in MOFs, demonstrating an overall positive effect when they are ‘held’ inside the MOFs’ structure.

**Fig. 26:**
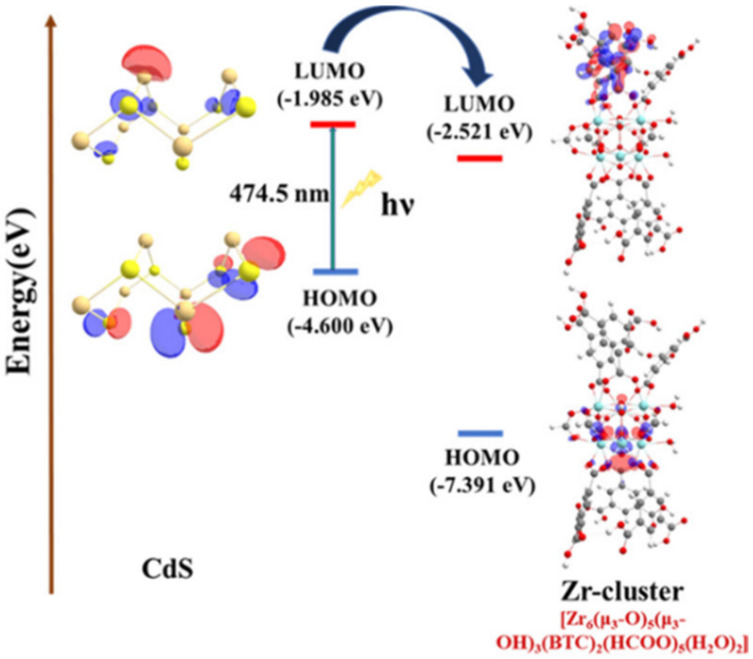
HOMO–LUMO energy positioning (eV) of the CdS-photosensitizer and Zr^4+^ cluster as catalysts for HER. The photoinduced electron transfer (PET) (H atoms have been omitted for clarity) is also illustrated. Reprinted with permission from ref. 7. Copyright 2022 American Chemical Society.

The confinement effect of CdSNPs within MOF pores was shown to dramatically improve photocatalytic HER (section 3).^7^ The proximity of nanoparticles to the MOF metal clusters allowed for a shorter electron transfer route – from the LUMO of CdS to the empty LUMO of the Zr^4+^ cluster – making the latter an energetically feasible catalytic centre for HER (Fig. 26). CdS_4_@MOF-808, containing 3.56% CdS, demonstrated a 60-fold increase in photocatalytic activity compared to CdS/MOF-808 due to the agglomeration of CdSNPs on the surface of the MOF. This enhanced catalytic ability of CdS_4_@MOF-808 was attributed to several factors: increased current density, improved separation of photo-induced electron–hole pairs, lower charge-transfer resistance, and quenching of photoluminescence (PL) with shortened PL lifetime.

Similarly, the effect of the reactants’ transport pathway in a catalytic reaction on Fe_3_O_4_@MIL-100(Fe)–Pt was studied for the reduction of *p*-nitrophenol (*p*-NP) to *p*-aminophenol (*p*-AP).^52^ Both the parent MOF and Fe_3_O_4_ exhibited no catalytic activity; however, Fe_3_O_4_@MIL-100(Fe)–Pt showed complete catalytic conversion in all samples. The fastest conversion, within 5 min, was achieved by Fe_3_O_4_@MIL-100(Fe)–Pt(30), where PtNPs were deposited on the 30^th^ layer of MIL-100, meaning the NPs resided closer to the MOF surface than in other catalyst samples (section 2.2). This sample exhibited the greatest *pseudo*-first-order kinetics at 0.79 min^−1^, which was more than twice and nearly five times greater than Fe_3_O_4_@MIL-100(Fe)–Pt(20) and Fe_3_O_4_@MIL-100(Fe)–Pt(10), respectively.^52^ This study concluded that shorter pathways and higher loading led to the enhancement of reactivity.

To expand on the previous study, the MOF matrix could act as a sieve, allowing the diffusion of specific reactants through to the active site.^51^ A Pt@ZIF-8 composite synthesised *via* the “bottle-around-ship” method, as discussed in section 2.2, was studied and used as the catalyst for liquid-phase hydrogenation of *n*-hexane or cyclooctene. As a result of the shortened diffusion distance to the active site and the sieving effect, Pt@ZIF-8-‘sur’, with PtNPs located within the MOF layer, exhibited selectivity for linear-*n*-hexane and a three-fold higher activity compared to Pt@ZIF-8-‘in’, with PtNPs located adjacent to the ZnO nanorod. This result was further validated by comparing the catalytic activity of Pt@ZIF-8 to bare ZnO@ZIF-8 and Pt@carbon nanotubes (CNT). The bare MOF composite showed no hydrogenation activity, while Pt@CNT showed higher activity yet no selectivity.

Guo *et al*. synthesised flower-like NH_2_–UiO-68 embedded with PtNPs as photocatalysts for CO_2_ reduction (section 2.3).^54^ They found that the different locations of PtNPs, whether inside or on the exterior of the MOFs, affected the recombination of photogenerated electron–hole pairs. PL emission spectroscopy revealed that electron transfer was both efficient and faster in Pt@NH_2_–UiO-68 than NH_2_–UiO-68, which also exhibited the highest transient photocurrent response. This was attributed to the influence of the embedded PtNPs on the charge separation efficiency. Furthermore, EIS analysis showed low resistance during the charge transfer process. With these improved electronic properties, greater electron mobility and reduced electron–hole recombination were expected. The photocatalytic CO_2_ reaction results aligned with this observation as the PtNPs significantly enhanced the activity compared to the unloaded NH_2_–UiO-68. Furthermore, the photocatalytic activity of Pt@NH_2_–UiO-68 was three times higher than for Pt/NH_2_–UiO-68, showing that the intimate contact of PtNPs within the MOF structure facilitated rapid charge transfer.^54^

**Fig. 27:**
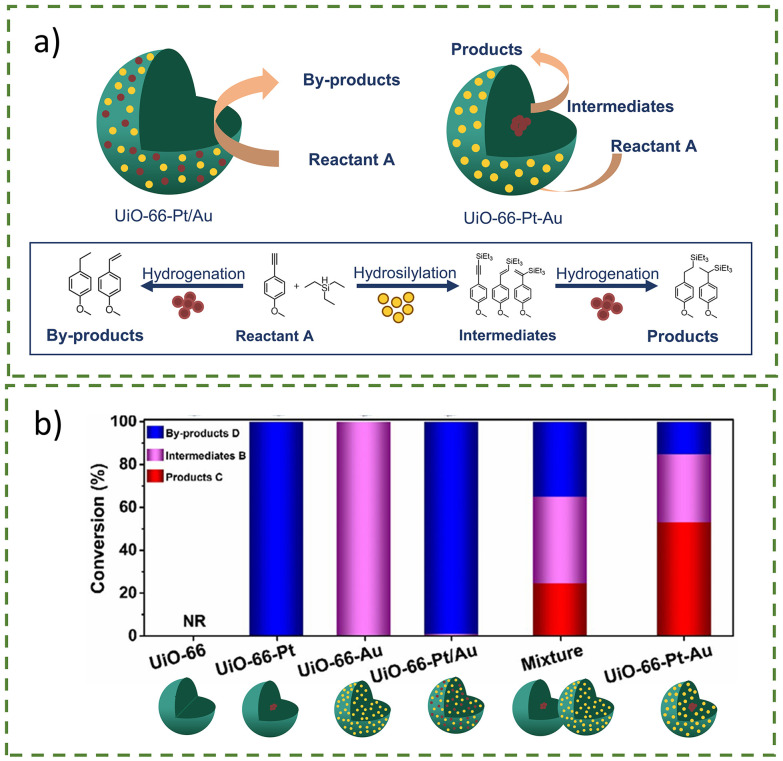
(a) Scheme of hydrosilylation–hydrogenation tandem reaction catalysed by UiO-66–Pt–Au, or the formation of by-products due to the favourable hydrogenation reaction with UiO-66–Pt/Au. (b) Catalytic performance of various precursors and catalysts in the study based on conversion to by-products, intermediates, or products. Adapted with permission from ref. 3. Copyright 2022 Springer Nature.

Another area that has been explored more recently is the combination of different metal nanoparticles within a MOF for tandem catalysis, exploiting the different catalytic nature of each metal nanoparticle. Pan *et al*. investigated the spatial distribution of AuNPs and PtNPs in UiO-66 as catalysts for tandem hydrogenation–hydrosilylation reactions (Fig. 27a).^3^ UiO-66-Pt–Au was synthesised by *in situ* encapsulation of PtNPs within UiO-66, followed by impregnation and reduction of AuNPs. TEM analysis showed PtNPs concentrated in the core area, while AuNPs were dispersed homogeneously on the shell. This spatial distribution proved essential for catalysis, as UiO-66–Pt–Au outperformed other reported catalysts (Fig. 27b). The proposed mechanism begins with the hydrosilylation on the AuNP shell, followed by diffusion of the intermediates and the formation of the products, with PtNPs facilitating the hydrogenation reaction (Fig. 27a). However, the selectivity of the final saturated silane products was only 53% due to the ability of some reactants to diffuse directly to PtNPs, bypassing AuNPs. In contrast, UiO-66–Pt/Au, with both AuNPs and PtNPs randomly distributed throughout UiO-66, favoured the hydrogenation reaction, forming by-products. Therefore, this spatial distribution design of nanoparticles is crucial for controlling tandem catalysis.

## 6. Catalytic applications of NP-MOF composites

This section discusses recent catalyst advancements in the past five years related to nanoparticles with Zr-based MOFs. In addition to their catalytic reactivity, Zr-based MOF composites have been explored for biological applications,^93^ wastewater treatments,^94^ and as adsorbents.^4^ In many catalytic reactions, pristine MOFs exhibit low to negligible activity, pointing to the metallic nanoparticles hosted by the MOFs as the main active sites. Besides preventing nanoparticle aggregation and enhancing stability, some studies suggest that metal clusters of MOFs may also contribute to the overall catalytic performance of the NP-MOF composite.

### 6.1. CO_2_ conversion

The growing emission of carbon dioxide (CO_2_), a major greenhouse gas, has led to significant efforts focused on its capture and conversion. Despite its chemical stability, CO_2_ can be converted into useful and value-added chemical intermediates/products for fuel or pharmaceutical industries through various reactions under ambient conditions or using electrochemical or photochemical methods.^95,96^

The hydrogenation reactions of CO_2_ to methanol or methane have been extensively studied using NP-MOF composites such as UiO-67-Pt^6^ and Ni@MOF-545^33^ for methanol, or Ni@UiO-66^97^ for methane. Several factors are repeatedly reported to influence the overall catalytic activity, including defect concentration, linker functionalisation, metal at the metal cluster, surface area, and nanoparticle size. For example, the defect density in the UiO-67-Pt system was changed through the bpdc healing procedure, where coordinated benzoic acids were replaced with the linkers. With the lower number of defects, indicating fewer open Zr-sites in the PtNPs-MOF interface of the UiO-67–Pt, the formation of methanol and methane decreased. On the other hand, carbon monoxide formation was not affected because the catalysis took place on the surface of PtNPs.^98,99^ Furthermore, hydration of the Zr node inhibited methane formation, yet assisted in the desorption of synthesised methanol from the catalyst, hence increasing methanol selectivity.

Within this family of MOFs, Kobayashi *et al*. studied various CuNP-covered MOFs, which were tested as catalysts for CO_2_ hydrogenation to methanol.^100^ Among the different supports for CuNPs–Al_2_O_3_, ZIF-8, MIL-101, and UiO-66–Cu/Zr–UiO-66 exhibited the highest CO_2_ conversion activity. Unlike the previous example, defects at the Zr_6_ nodes did not significantly affect the catalyst's reactivity. However, MOF ligand functionalisation (–NH_2_, –COOH) or replacement of Zr with Hf, led to a three-fold increase in reactivity for Cu/Zr–UiO-66–COOH and Cu/Hf–UiO-66. This enhancement in reactivity was attributed to the charge transfer between CuNPs and the MOF, which could be observed by XPS, with a greater shift in binding energy for Cu 2p. Therefore, as charges are transferred from CuNPs, the resulting cationic Cu species could stabilise formate, an intermediate of the hydrogenation reaction, enhancing the CO_2_ conversion activity.^100^

**Fig. 28:**
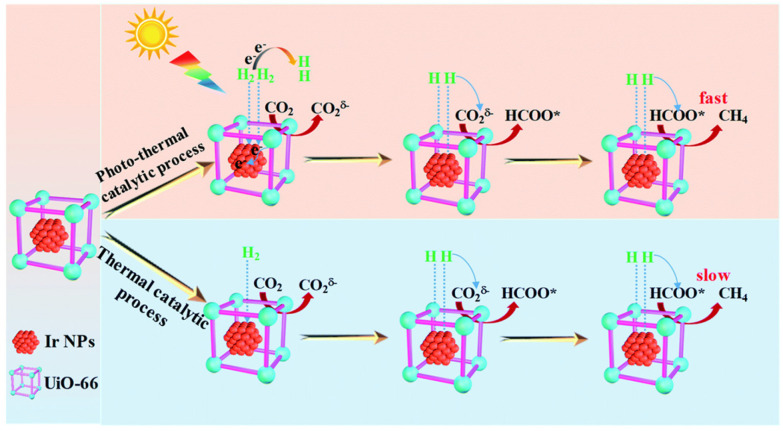
Proposed mechanism using Ir@UiO-66 for the thermal and photo-thermal catalytic Sabatier reaction process. Reprinted with permission from ref. 101. Copyright 2022 Royal Society of Chemistry.

For methane production, two pathways are possible: reverse water gas shift (RWGS) followed by CO hydrogenation, or the *HCOO pathway. For example, Ir@UiO-66 demonstrated high photocatalytic activity for CO_2_ methanation, with charge separation and transfer promoted by the IrNP core, as evidenced by the higher photocurrent response and lower charge transfer resistance.^101^ Under elevated temperatures, methane production increased to 19.9 mmol g_cat_^−1^ h^−1^ with 95% CH_4_ selectivity. Under additional illumination at the same temperature, the methane production rate was increased sevenfold. Thus, the enhanced activity was attributed to the synergistic contribution of IrNPs to dissociate H_2_, and UiO-66 to adsorb/activate CO_2_ (Fig. 28). Similarly, highly dispersed 2 nm NiNPs in UiO-66 achieved a 57.6% conversion with 100% selectivity for methane.^97^

Furthermore, Wang *et al*. demonstrated the difference in activity for plasmonic-induced photothermal catalysis for CO_2_ hydrogenation using bimetallic AuPt alloy NPs and Au@Pt core–shell NPs encapsulated in UiO-66–NH_2_.^102^ The CO_2_ hydrogenation reaction was conducted in vapour–solid mode at 150 °C under irradiation with a 300 W Xe lamp for 4 h. Overall, AuPt@UiO-66–NH_2_ showed the highest activity, which the authors proposed was due to a combination of effects, including the localised surface plasmon resonance (LSPR) effect of AuNPs, the catalytic role of PtNPs, and MOF encapsulation. The LSPR effect of AuNPs generated ‘hot electrons’, which indirectly reduced the activation energy for CO_2_ conversion. Differences in the metal arrangement inhibited the LSPR effect of gold in core–shell Au@PtNPs, while for the alloyed NPs, it had a synergistic effect. In terms of MOF encapsulation, UiO-66–NH_2_ acted as a microreactor, improving CO_2_ adsorption due to its high surface area and alkalinity. Overall, the AuPt@UiO-66–NH_2_ exhibited a conversion rate of 1451 μmol g_meta_^−1^ h^−1^ for CO and 137 μmol g_meta_^−1^ h^−1^ for CH_4_, with 91% selectivity towards CO.^102^

CO_2_ can also be converted into cyclic carbonates, offering a promising route for CO_2_ utilisation. Cu@UiO-66–NH_2_ demonstrated high catalytic activity, achieving 85% conversion for CO_2_ coupling with propargylic alcohols and complete transformation of propargylic amines with CO_2_.^103^ The CuNP/MOFs exhibited a yield of 88% for the one-pot synthesis of 2-oxazolidinones comprising of CO_2_, aliphatic amine, phenylacetylene, and acetone. The synergy between CuNPs and UiO-66–NH_2_ facilitated CO_2_ activation and enhanced the catalytic performance, with 100% product selectivity and stability maintained over six cycles. CO_2_ fixation into cyclic carbonates was also studied using silver-based catalysts (see section 3).^72,76^ Ag@Zr–DMBD, in combination with tetrabutylammonium bromide (TBAB), a co-catalyst, also showed an improved catalytic activity for the reaction of epichlorohydrin (EPH) with CO_2_, yielding >99% conversion at 60 °C.^72^ For this catalyst, both Ag and Zr were active centres responsible for the reaction by synergistically binding to the electron-rich oxygen atom of the terminal epoxide.

The use of AgNPs in Zr-based MOFs has also been reported by Aparna *et al*. to catalyse A^3^ coupling reactions and the electrochemical reduction of CO_2_.^73,104^ In the latter reaction, Ag@UiO-66–SH showed enhanced selectivity towards CO formation, achieving a maximum faradaic efficiency of 74% at −1.1 V. In contrast, CO_2_ reduction in the presence of pristine UiO-66–SH only yielded hydrogen gas. This catalyst displayed one of the highest partial current densities for CO generation at 218 A g^−1^ at −1.1 V, outperforming other silver-based catalysts.^104^ Furthermore, the CO generation was stable for 10 hours, yielding stable faradaic efficiency.

### 6.2. Hydrogen evolution reaction

The hydrogen evolution reaction (HER) is a clean and renewable method for synthesising hydrogen molecules. Recently, Mandal *et al*. reported a series of nanoparticles encapsulated in Zr-based MOFs as electrocatalysts for HER.^71,105,106^ Functionalisation of UiO-66 and NU-1000 with thiol or amino groups stabilised the corresponding nanoparticles, resulting in their homogeneous dispersion and enhanced reactivity for HER. Under identical electrochemical conditions using 0.5 M sulfuric acid, similar trends in HER performance were observed for AgNPs encapsulated in NU-1000, Cu@NU-1000–NH_2_, and Pt@UiO-66–SH.^71,105,106^ Among the silver-loaded NU-1000 samples, 0.25 Ag–NU (prefix number refers to the AgNO_3_ millimoles) exhibited the highest reactivity, showing a reduced overpotential of 165 mV in an acidic medium at 10 mA cm^−2^ and a Tafel slope of 53 mV dec^−1^.^71^ Stability tests confirmed that 0.25 Ag–NU remained stable over 24 h with a turnover frequency (TOF) of 20.2 s^−1^. Cu@NU-1000–NH_2_ exhibited an overpotential of 158 mV at 10 mA cm^−2^, a Tafel slope of 105 mV dec^−1^, 45 h stability, and a faradaic efficiency of 91.55%.^105^ Lastly, Pt@UiO-66–SH exhibited a lower overpotential of 57 mV at 10 mA cm^−2^, a Tafel slope of 75 mV dec^−1^ with a high TOF value of 389.37 s^−1^, and 30 h stability.^106^ The enhancement in HER catalytic activity is attributed to the presence of metallic nanoparticles, which increased the electrochemically active surface area and the number of active sites for H_2_ adsorption. Moreover, Ding *et al*. investigated the HER performance of RhRu alloy NPs in UiO-66–NH_2_ at different pH values.^107^ The optimised Rh_50_Ru_50_@UiO-66–NH_2_ exhibited the best performance, with an overpotential of 77 mV at 10 mA cm^−2^, a Tafel slope of 79 mV dec^−1^, outperforming Pt/C, a TOF of 0.273 s^−1^, and stability over 3000 cycles. HER activity was found to be superior in acidic compared to neutral and alkaline conditions.

### 6.3. Conversion of organic compounds

Chen *et al*. reported the catalytic activity of Pd@UiO-66-X for the hydrogenation of benzoic acid to cyclohexane carboxylic acid.^108^ Using MOF linkers with different moieties, including –H, OMe, NH_2_, or 2OH–PdNPs were encapsulated and dispersed within the MOF, with an average size of 1.1 nm and Pd loading between 2.13–2.84 wt%. The catalytic activity followed the order of 2OH > NH_2_ > OMe > H, with Pd@UiO-66–2OH exhibiting 14-fold higher activity than Pd@UiO-66. The trend was explained by the electron-donating capabilities of the functional group, where stronger donors facilitated greater catalytic efficiencies through reduced substrate adsorption, as predicted by the Sabatier principle.^109^

Redfern *et al*. investigated the hydrogenation of acetylene using Cu–SIM–NU-1000 in a fixed-bed reactor.^10^ Reduction of Cu oxo-clusters into CuNPs, followed by cofeeding the system with acetylene/H_2_ gas at a 1 : 1 ratio, yielded ethylene, ethane, and C_4_ byproducts (1-butene and 1,3-butadiene). The catalyst exhibited high selectivity for ethylene, with less than 0.5 mol% of ethane and C_4_ byproducts in the product stream. In later work, the group compared smaller CuNPs in NU-901 and NU-907 to the same catalytic reaction.^92^ The hydrogenation of acetylene using both CuNPs@NU-901 and CuNPs@NU-907 yielded ethylene, 1-butene, and 1,3-butadiene without ethane. By comparing the TOF values, the performance of CuNPs@NU-901 and CuNPs@NU-907 was significantly lower than CuNPs@NU-1000 at 13.5 ± 0.8, 7.7 ± 0.2, and 100 ± 20 h^−1^, respectively.^10,92^ This finding disproves their hypothesis that the smaller CuNPs would enhance the catalytic efficiencies.

Formic acid is an organic molecule that can be converted to hydrogen by dehydrogenation reactions, providing an effective and non-toxic method for hydrogen production.^110,111^ Ding *et al*. reported that PdAuNPs encapsulated in UiO-66–NH_2_ synthesised *via* DSM (Pd_0.8_Au_0.2_/UiO-66-D) outperformed the catalyst synthesised by the impregnation method (Pd_0.8_Au_0.2_/UiO-66-S).^30^ Pd_0.8_Au_0.2_/UiO-66-D released 112 mL of gas within 25 min (initial TOF of 3122 h^−1^), while Pd_0.8_Au_0.2_/UiO-66-S released 110 mL of gas within 5 h (initial TOF of 202 h^−1^). The greater activity was attributed to the smaller particle size of PdAuNPs in Pd_0.8_Au_0.2_/UiO-66-D (1.4 nm) compared to Pd_0.8_Au_0.2_/UiO-66-S (5.6 nm).^30^ Further optimisation led to the development of PdAu/UiO-66-(NH_2_)_2_, containing diamine functionalisation, improving the stabilisation of nanoparticles due to increased hydrophilicity.^112^ Pd_0.8_Au_0.2_/UiO-66-(NH_2_)_2_ synthesised *via* DSM yielded highly dispersed 1.1 nm PdAuNPs with enhanced dehydrogenation performance, producing H_2_ : CO_2_ at a 1 : 1 ratio, with gas formation at 111 mL within 20 min and a TOF value of 3660 h^−1^ at 323 K. Recyclability of the composite was also observed over seven runs with no significant difference in activity. The importance of the amine group was studied by comparing the activity of Pd_0.8_Au_0.2_/UiO-66-(NH_2_)_2_ and Pd_0.8_Au_0.2_/UiO-66. It was found that the amine functionality enhanced PdAu performance by producing smaller nanoparticles, leading to more active surfaces and inducing higher basicity in the framework, preventing the dimerisation of formic acid.^112^

**Fig. 29:**
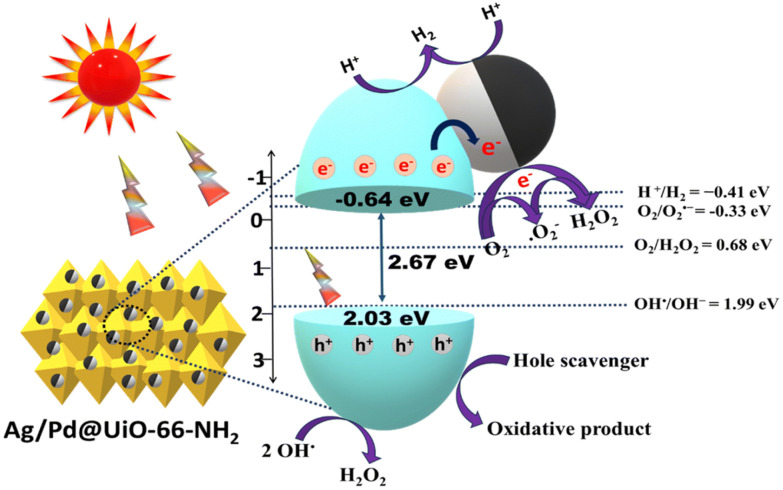
Schematic representation of the mechanistic pathway for H_2_O_2_ and H_2_ evolution using Ag/Pd@UiO-66–NH_2_ as a photocatalyst, including the ability of Ag/PdNPs to act as electron traps to limit electron pair recombination. Reprinted with permission from ref. 21. Copyright 2024 Royal Society of Chemistry.

In another work, Dash *et al*. reported the ability of Ag/Pd@UiO-66–NH_2_ to act as a photocatalyst for H_2_O_2_ and H_2_ production, showing a four-fold increase in production capacity compared to UiO-66–NH_2_ (Fig. 29).^21^ Despite the adsorption–reduction method yielding Ag/PdNPs on the surface of the MOF, Ag/Pd@UiO-66–NH_2_ with 1 : 2 Ag : Pd ratio exhibited increased reactivity for both H_2_O_2_ and H_2_ production at a rate of 39.4 mmol h^−1^ and 448.2 mmol h^−1^, respectively. This enhancement of reactivity was attributed to Ag/PdNPs improving the photon trapping ability, better exciton segregation tendency, and creating suitable band energies (valence band = VB = 2.03 eV and conduction band = CB = 0.64 eV). In addition to the greater light adsorption, due to the LSPR effect of AgNPs, Ag/PdNPs acted as electron traps, creating the Schottky barrier that suppresses the recombination of the electron pairs. Since the composite exhibited suitable VB and CB potentials for electron transfer, the catalytic activity was significantly improved.

The reduction of H_2_O_2_ is also an important process in producing reactive oxygen species (ROS), a crucial oxidant for pollutants.^113^ Yin *et al*. developed Fe_3_O_4_@UiO-66 and Fe_3_O_4_@UiO-67 as catalysts for a Fenton-like reaction involving bisphenol A (BPA) as a model contaminant.^94^ H_2_O_2_/Fe_3_O_4_@UiO-66 exhibited four-fold greater activity compared to the H_2_O_2_/Fe_3_O_4_ system due to the formation of a dual active centre and charge transfer between Fe and Zr. Due to the electron-deficient nature of the biphenyl-4,4′-dicarboxylic acid linkers in UiO-67, the charge density of the Zr nodes is less than the Zr nodes in UiO-66, leading to reduced charge transfer from Zr to Fe(iii). This difference is reflected in the lower *pseudo*-first-order rate constant for BPA degradation in H_2_O_2_/Fe_3_O_4_@UiO-67 (0.028 s^−1^) compared to H_2_O_2_/Fe_3_O_4_@UiO-66 (0.046 s^−1^). Therefore, it was concluded that UiO MOFs and Fe_3_O_4_NPs acted as dual active centres, synergistically mediating and regulating the charge density relationship between Zr and Fe, influencing the rate of H_2_O_2_ activation and degradation of BPA.^94^

**Fig. 30:**
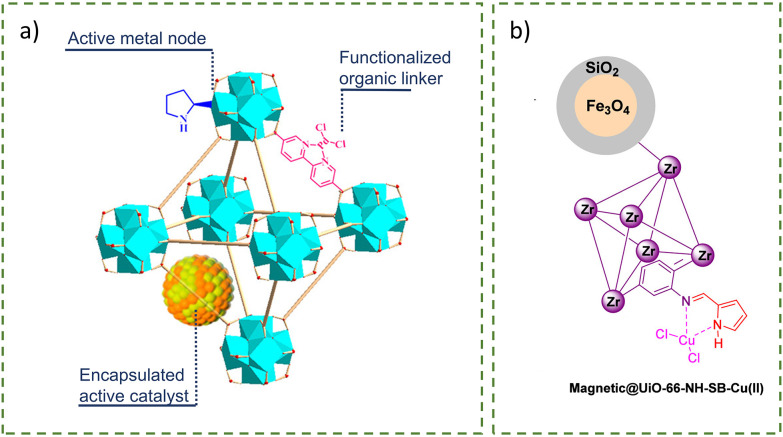
Schematic representation of multifunctional MOF composites, (a) novel trifunctional chiral heterogeneous MOF catalyst. Adapted with permission from ref. 114. Copyright 2023 American Chemical Society, (b) magnetic@UiO-66–NH–SB–Cu(ii). Reprinted with permission from ref. 115. Copyright 2025 American Chemical Society.

Furthermore, Cheng *et al*. reported the synthesis of a novel trifunctional chiral catalyst, CuCo@UiO-67–Pd(ii)–l-Pro, with three active catalysts incorporated into the UiO-67 scaffold by multiple modifications to the MOF structure (Fig. 30a).^114^ The catalyst was used for the asymmetric three-step sequential catalytic oxidation of aromatic alcohols, Suzuki coupling, and asymmetric aldol reaction, with higher catalytic efficiencies than the individual components at 95%, 96%, and 72% yield, respectively. The asymmetric aldol condensation reaction also showed enantioselectivity at 73% ee_anti_. In addition, the catalyst is stable under reaction conditions, with no structural change observed after five successive catalytic cycles and no leaching detected. The enhanced activity was due to the synergistic effect of the individual components; CuCoNPs enhanced the aromatic oxidation, and the Pd(ii) grafted linker with the l-proline ligated Zr cluster was responsible for Suzuki coupling/asymmetric aldol reactions.^114^

In 2025, magnetic@UiO-66–NH–SB–Cu(ii) catalyst obtained through the “bottle-around-ship” method was reported by Manafi Moghadam *et al*., for a one-pot multicomponent reaction to synthesise 2-amino-3-cyano-4*H*-chromene derivatives (Fig. 30b).^115^ This work shows the combination of enhanced catalytic activity from the Cu(ii) species incorporated by the Schiff base moiety, with the reusability of the magnetic component, thus allowing a good separation.

Other examples showcasing the catalytic activities of NP@Zr-MOFs for C–C reactions include A^3^ coupling reactions to obtain propargylamine^73,78^ and cross-coupling (Suzuki, Heck, and Sonogashira) reactions.^77^ Oxidation and reduction reactions are also possible, such as the oxidation of toluene,^116,117^ oxidation of aromatic alcohols into aldehydes,^75^ oxidation of hydrazine.^118^

## 7. Conclusions

Nanoparticle-metal–organic framework (NP-MOF) composites have emerged as a promising class of materials that integrate the tuneable porosity and structural versatility of MOFs with the high catalytic activity of metal nanoparticles. This review discusses in detail the versatility of MOFs for modification, allowing them to serve as hosts for metal nanoparticles, preventing nanoparticle aggregation and loss of catalytic activity. The preparation of NP-MOF composites is divided into five major categories, with “ship-in-bottle”, “bottle-around-ship”, and “one-pot reaction” being the more commonly used methods. Depending on the application of the composite, all of the mentioned strategies are capable of synthesising NP@MOF or NP/MOF composites. Therefore, it is critical to consider the design of the MOF host to influence the spatial location of nanoparticles. It cannot be determined that having nanoparticles on the surface of the MOF is disadvantageous over inside the MOF, but the general conclusion from the catalytic studies has shown that NP@MOFs exhibited higher activity than their NP/MOF counterparts for several electrochemical and photochemical reactions. Further, zirconium-based NP-MOF systems were especially considered, given their superior chemical stability and potential for catalytic applications such as CO_2_ conversion, hydrogen evolution reactions, and organic transformations.

While significant progress has been made in the development and application of NP-MOF composites for catalysis, several challenges remain that hinder their widespread implementation:

I. The scalability of NP-MOF composite formation is limited by the method adopted, which could lead to the use of expensive metal precursors or an excessive amount of solvent required compared to the reactant.

II. To achieve precise control over the spatial localisation of nanoparticles within MOFs, further investigation into the double solvent method, atomic layer deposition, coordination-assisted templating, or new state-of-the-art techniques could be optimised to accurately incarcerate NPs in the desired location.

III. Detection of spatial distribution of nanoparticles is a challenging aspect because the detailed characterisation of the nanoparticles’ location within a MOF composite often requires advanced characterisation techniques, such as electron tomographic reconstruction, different envelope density (DED) analysis, and positron annihilation.

IV. The catalytic activity of NP-MOFs is often limited by mass transport and diffusion constraints, particularly when bulky reactants or intermediates are present. Although MOFs possess well-defined porosity, their rather small pore sizes can limit or even prevent accessibility to encapsulated nanoparticles, reducing overall reaction rates. As such, strategies to address this problem, such as hierarchical structuring, incorporating meso- or macropores, or defect-engineered MOFs, need to be further developed.

V. Structural stability and durability of MOFs to remain intact under harsh catalytic conditions need to be further investigated and studied to ensure long-term structural integrity.

VI. Leaching of metals into the reaction medium is one of the major concerns when utilizing heterogeneous catalysts such as NP-MOFs, as this can affect product purity and reproducibility. Furthermore, the leaching of toxic metals such as Pt or Pd can result in a cost-intensive product purification.

Given the rapid advancements in materials science and catalysis, significant opportunities remain for the further development of NP-MOF composites as heterogeneous catalysts, extending their applicability beyond the reactions discussed in this review. Future efforts should prioritise the spatial control of the NPs, but also the electronic interactions that result from these composite structures. In this context, despite the extensive use of thiol functional groups, their effect on the electron density at the NP surface and subsequent catalytic activity remains unexplored. Furthermore, the metal NP loadings to form the MOF composites are often very high; however, the resulting effects on the porosity, diffusion rates, and stability are often not considered. We believe that in order to predict these properties, a combination of both *in situ* characterisation methods (*e.g.*, X-ray absorption spectroscopy (XAS) and operando IR) and computational modelling, especially for popular reactions such as electrocatalytic CO_2_ reduction, needs to be utilised. Moreover, we have noticed that there are few reports comparing catalytic metrics (*e.g.*, turnover frequency, site-normalised activity) across different functionalisation strategies. Therefore, we believe that standardised metrics and mechanistic probes will greatly improve the understanding of structure–activity relationships. The insights presented herein are expected to stimulate innovative approaches for enhancing the stability, efficiency, and scalability of NP-MOF systems, ultimately facilitating their translation into industrially relevant catalytic processes.

## Conflicts of interest

There are no conflicts to declare.

## Data Availability

No primary research results, software or code have been included and no new data were generated or analysed as part of this review.
